# AMD1 promotes breast cancer aggressiveness via a spermidine-eIF5A hypusination-TCF4 axis

**DOI:** 10.1186/s13058-024-01825-6

**Published:** 2024-04-23

**Authors:** Ruocen Liao, Xingyu Chen, Qianhua Cao, Longchang Bai, Chenglong Ma, Zhijun Dai, Chenfang Dong

**Affiliations:** 1 Department of Breast Surgery, First Affiliated Hospital, Zhejiang University School of Medicine, 310058 Hangzhou, China; 2 Department of Pathology and Pathophysiology, Department of Surgical Oncology (breast center), Key Laboratory of Cancer Prevention and Intervention, The Second Affiliated Hospital, Ministry of Education, Zhejiang University School of Medicine, 310058 Hangzhou, China; 3 Zhejiang Key Laboratory for Disease Proteomics, Zhejiang University School of Medicine, 310058 Hangzhou, China

**Keywords:** AMD1, Basal-like breast cancer (BLBC), eIF5A, Hypusination, TCF4

## Abstract

**Background:**

Basal-like breast cancer (BLBC) is the most aggressive subtype of breast cancer due to its aggressive characteristics and lack of effective therapeutics. However, the mechanism underlying its aggressiveness remains largely unclear. S-adenosylmethionine decarboxylase proenzyme (AMD1) overexpression occurs specifically in BLBC. Here, we explored the potential molecular mechanisms and functions of AMD1 promoting the aggressiveness of BLBC.

**Methods:**

The potential effects of AMD1 on breast cancer cells were tested by western blotting, colony formation, cell proliferation assay, migration and invasion assay. The spermidine level was determined by high performance liquid chromatography. The methylation status of CpG sites within the AMD1 promoter was evaluated by bisulfite sequencing PCR. We elucidated the relationship between AMD1 and Sox10 by ChIP assays and quantitative real-time PCR. The effect of AMD1 expression on breast cancer cells was evaluated by in vitro and in vivo tumorigenesis model.

**Results:**

In this study, we showed that AMD1 expression was remarkably elevated in BLBC. AMD1 copy number amplification, hypomethylation of AMD1 promoter and transcription activity of Sox10 contributed to the overexpression of AMD1 in BLBC. AMD1 overexpression enhanced spermidine production, which enhanced eIF5A hypusination, activating translation of TCF4 with multiple conserved Pro-Pro motifs. Our studies showed that AMD1-mediated metabolic system of polyamine in BLBC cells promoted tumor cell proliferation and tumor growth. Clinically, elevated expression of AMD1 was correlated with high grade, metastasis and poor survival, indicating poor prognosis of breast cancer patients.

**Conclusion:**

Our work reveals the critical association of AMD1-mediated spermidine-eIF5A hypusination-TCF4 axis with BLBC aggressiveness, indicating potential prognostic indicators and therapeutic targets for BLBC.

**Supplementary Information:**

The online version contains supplementary material available at 10.1186/s13058-024-01825-6.

## Background

S-adenosylmethionine decarboxylase proenzyme (AMD1) is a key enzyme that involved in the synthesis of polyamines, including spermine and spermidine [1]. AMD1 has been regarded as an oncogene in several cancers and a potential target for tumor therapy [1, 2]. However, the exact mechanism and function of AMD1 on tumor cells remains largely unclear. Polyamines are associated with multiple cellular processes, including differentiation and cell proliferation [1, 3]. Spermidine, a key AMD1-mediated downstream polyamine is a precursor of hypusine, which is involved in the lysine-50 (K50) residue hypusination of eukaryotic initiation factor 5 A isoform 1 (eIF5A). eIF5A is the only protein known to be activated by hypusination [1]. Ribosome-bound, hypusinated eIF5A interacts with the peptidyltransferase center of the ribosome, orienting and stabilizing the CCA end of the peptidyl tRNA to allow protein synthesis through preventing ribosomal stalling in the translation of mRNAs encoding polyproline motifs (Pro-Pro motif) and certain other amino acid combinations [1, 3]. Recent studies have shown that eIF5A expression is upregulated in multiple cancers, including breast cancer [4–6]. A few studies have reported that hypusinated eIF5A promotes translation of RhoA and MYC in tumor cells [4, 5]. However, the regulation of the hypusination and target genes associated with eIF5A hypusination are still inadequate, which has prevented the development of novel anticancer drugs.

Transcription factor 4 (TCF4) is a key transcription factor of the TCF/LEF family, which activates target gene transcription by binding to the E-box through the SOX-like HMG domain. There are four members of the family, including TCF1, LEF1, TCF3 and TCF4, which are involved in the Wnt signaling pathway [7, 8]. In breast cancer, the activation of Wnt/TCF signaling is considered to be responsible for breast cancer proliferation, immune microenvironment regulation, stemness maintenance, metastasis and therapeutic resistance [9]. In this study, we demonstrate that AMD1 expression is remarkably elevated in BLBC, a subtype that has poor clinical prognosis due to its aggressiveness and lack of effective therapeutics [10]. AMD1 activates TCF4 translation by enhancing spermidine production and eIF5A hypusination of breast cancer cells, promoting breast cancer aggressiveness.

## Materials and methods

### Plasmids and antibodies

Human AMD1 gene was amplified from SUM159 cDNA library, and sub-cloned into pLVX-puro. Human Sox10 and eIF5A gene were amplified from MDA-MB231 cDNA library and subcloned into pLVX-Puro and.

pLenti6/V5-D-TOPO® vector, respectively. Antibody against AMD1 was purchased from Proteintech (catalog no. 11052-1-AP). Antibody against FLAG was purchased from Sigma-Aldrich (catalog no. F3165). Antibody against β-actin was purchased from ABclonal (catalog no. AC038). Antibody against eIF5A was purchased from Abcam (catalog no. ab32443) and Hypusine antibody (catalog no. PABL-202) was purchased from Creative Biolabs. Antibody against TCF4 was purchased from EPITOMICS (catalog no. 2114-1). Goat Anti-Mouse IgG H&L (Alexa Fluor® 488) (catalog no. ab150113) and Goat Anti-Rabbit IgG H&L (Alexa Fluor® 555) (catalog no. ab150078) were purchased from Abcam.

### CRISPR-Cas9 genome editing and verification

The design of gRNA targeting human eIF5A was carried out using online tools from Zhang’s lab [11], then the target sequence was cloned into lentiCRISPR v2 plasmid (Addgene). LentiCRISPR v2 plasmid was cotransfected into HEK293T with the packaging plasmid psPAX2 and pMD2.G using Lipofectamine™ 3000 (Thermofisher) for virus production. Filtered viral supernatants were used for transfecting MDA-MB231, BT549 cells. Cells were selected using puromycin (300ng/mL), and single cell was seeded into 96-wells flat-bottom plates (Corning). Cells from single-cell derived clones were harvested, and DNA was extracted for genome editing verification. The gRNA primers used for CRISPR-Cas9 were: 5’- CACCG TGGCAAGCACGGCCACGCCA − 3’ (forward) and 5’- AAACTGGCGTGGCCGTGCTTGCCA C-3’ (reverse).

### Cell culture

All cells we used in this study were obtained from the American Type Culture Collection (Manassas, VA), where the cell lines were authenticated by STR profiling before distribution. MDA-MB231, SUM159, Hs578T and HEK293T cells were grown in Dulbecco’s modified Eagle’s Medium (DMEM)/F12 with 10% FBS. BT549 cells were grown in RPMI-1640 supplemented with 10% FBS. MDA-MB468 cells were cultured in Leibovitz’s L-15 medium with 10% FBS. All the cells were cultured and stored according to the instruction from the ATCC. For establishing stable transfectants with overexpression of AMD1, Hs578T and BT549 cells were transfected with pLVX-puro-AMD1; stable clones were selected using 300 ng/mL puromycin for 4 weeks. MDA-MB231 KO and BT549 KO cells were transfected with pLenti6/V5-eIF5A-WT, pLenti6/V5-eIF5A-K50R; stable clones were selected using 5 µg /mL Blasticidin for 4 weeks.

### Quantitative real-time PCR

Total RNA was extracted from cells by AG RNAex Pro Reagent (Accurate Biology) according to the manufacturer’s instructions. Reverse transcription was performed with the Evo M-MLV II Reverse Transcriptase (Accurate Biology). Real-time quantitative PCR (RT-qPCR) was performed using SYBR Green Premix Pro Taq HS qPCR Kit (Accurate Biology) according to the manufacturer’s protocol. Gene expression level was normalized to GAPDH level in respective samples as an internal control, and the results were performed with at least three independent experiments. The primers used for RT-qPCR were: 5’- CCCTGTTGAAGCTTGCTAGG-3’ (forward) and 5’-TGGGTACCCTTGGTGAGAAG-3’ (reverse) for AMD1; 5’-TGGACCGCACACCTTGGGACA-3’ (forward) and 5’-ACGCCCACCTCCTCCGACCT-3’ (reverse) for Sox10; 5’-GCTCCTCCGATTCCGAGG-3’ (forward) and 5’-TGTTAGAGACAATGTGT-3’ (reverse) for TCF4; 5’-GCTGCGAAGTGGAAACCATC-3’ (forward) and 5’-CCTCCTTCTGCACACATTTGAA-3’ (reverse) for cyclin D1; 5’-TGCACCACCAACTGCTTAGC-3’ (forward) and 5’-GGCATGGACTGTGGTCATGAG-3’ (reverse) for GAPDH.

### Colony formation assay and CCK-8 assay

Colony formation assay was performed using double-layer soft agar in 24-well plates with a bottom layer of 0.7% agar and a top layer of 0.35% agar. Different cells were seeded in 24-well plates and cultured in desired medium with proper cell counts at 37 °C, and the colonies were counted after cultivation for 21 ∼ 28 days. Approximately 1000 cells per well were cultured in a 96-well plate, and treated with 100 µL of medium containing 10 µL CCK-8 reagent (Yeasen). Following incubation at 37 °C, with 5% CO2 for 1 h, each well’s optical density was measured at 450 nm by iMark™ Microplate Absorbance Reader (Bio-Rad). All experiments were performed in triplicate.

### Methylation-specific PCR

To evaluate the methylation status of CpG sites within the AMD1 promoter, genomic DNA was extracted from each breast cancer cell line. 1 µg extracted DNA was bisulfite converted using the EpiTect Bisulfite Kit (Qiagen) according to the manufacturer’s instructions. Methylation-specific PCR reaction was performed using 2×EpiArt HS Taq kit (Vazyme) with the following nested primers:

Forward methylated primer: 5’- GATTGTATAGAGAAGTTAACGGGT-3’, Reverse methylated primer: 5’- GTTATAAAACTTCCAATCGACTAAACG − 3’.

Forward unmethylated primer: 5’- GGATTGTATAGAGAAGTTAATGGGT-3’, Reverse unmethylated primer: 5’- ATTATAAAACTTCCAATCAACTAAACACC − 3’.

20 ng bisulfite-converted DNA was used as the template. The PCR steps were as follows: 95 ℃ for 5 min, 29 cycles of 95 ℃ for 30 s, 58 ℃ for 30 s and 72 ℃ for 30 s, followed by a final extension at 72 °C for 5 min. The PCR product was analyzed by gel electrophoresis, and visualized images were captured.

### Luciferase reporter assay

Experiments were performed as described previously [12, 13]. All experiments were performed three times in triplicate.

### Chromatin immunoprecipitation (ChIP)

ChIP assays were performed as described previously [12, 13]. The following primers were used for ChIP assays: 5’- AAGGCTTCCCAAGGTGTTCC − 3′ and 5’- CTCTGCCAGATGACTGTGGG-3′ for the AMD1 promoter. The cells were prepared to perform ChIP assay with the Imprint ChIP Kit (Sigma) according to the manufacturer’s instructions and as described recently [12].

### Western blot analysis

Cell lysates was prepared using RIPA buffer containing protease inhibitors (Roche), and protein concentration was measured using Bradford assay kit (Fude Biological Technology). Adjusted protein samples were mixed with loading buffer and electrophoresed on 12% sodium dodecyl sulfate-polyacrylamide (SDS-PAGE) gels. After electrophoresis, proteins were transferred to polyvinylidene fluoride (PVDF) membranes. The membranes were blocked with 5% fat-free milk for 1 h at room temperature. Blocked membranes were incubated with indicated diluted primary antibody following manufacturer’s recommendations and gently shaking at 4 °C overnight. The blots were visualized using ECL assay after incubation with secondary antibody on the next day.

### HPLC

The cells were washed twice with PBS and suspended in Lysis Buffer, and then supernatants were collected by centrifugation. 800 µl supernatants were mixed thoroughly with 500 µl Sodium hydroxide solution(2 M) and 10 µl Benzoyl chloride into a tube, and then incubated in water bath for 30 min at 40℃. Add 2.0 mL of saturated sodium chloride solution to abort the reaction. Following ether extract, evaporate the upper ether layer to dryness under vacuum, and suspend the residue in methanol. After filtering, supernatants were subjected to HPLC system using C18 column (4.6 × 250 mm,5 μm). Chromatography was carried out with a methanol: water (55:45). The flow rate was 1 mL/min and the detection was performed at 234 nm.

### Tumorigenesis assay

Animal experiments were performed according to the approved procedures by the Institutional Animal Care and Use Committee at Zhejiang University. To determine the effect of AMD1 on in vivo tumorigenesis, female nude mice (5 ∼ 6 weeks old) were injected with 5 × 10^6^ exogenous AMD1 knockdown cells in the left flank and vector control cells in the right flank. Tumor formation was monitored every 2 ∼ 4 d for 4 weeks. Tumor size and weight were measured.

### Statistical analysis

Results were expressed as mean ± SD or SEM as indicated. Comparisons were made by one-way ANOVA or the two-tailed Student’s t-test. Correlations were determined by Pearson’s correlation and Spearman’s rank correlation test. Survival curves were plotted using the Kaplan-Meier method, and differences were measured by the log-rank test. In all statistical tests, *p* < 0.05 was considered statistically significant.

## Results

### AMD1 is upregulated in BLBC subtype

**Fig. 1:**
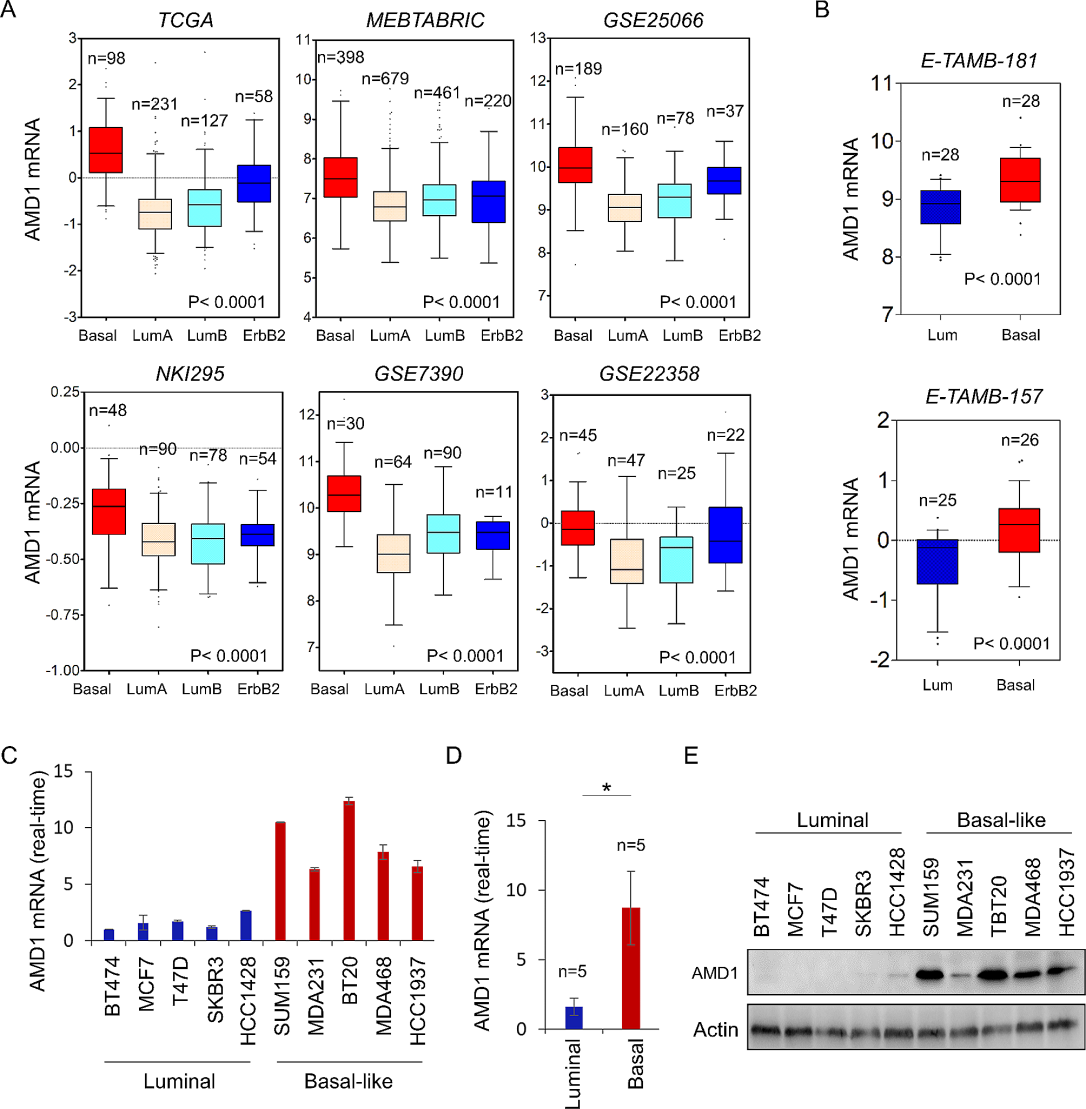
Elevated AMD1 expression was tightly correlated with BLBC. (**A**) Box-plots indicated AMD1 mRNA expression in four different subtypes of breast cancer from six datasets (TCGA, METABRIC, GSE25066, NKI295, GSE7390 and GSE22358). (**B**) Box-plots indicated AMD1 mRNA expression in BLBC and luminal cell lines from two different datasets (E-TAMB-181 and E-TAMB-157). Comparisons are made using the two-tailed Student’s t-test. (**C**, **D**) Expression of AMD1 mRNA was analyzed by quantitative real-time PCR in breast cancer cell lines (**C**), and the comparison between basal-like and luminal breast cancer cell lines from (**C**) was analyzed (**D**). Data are shown as mean ± SD based on three independent experiments. (**E**) Expression of AMD1 in cells from (**C**) was examined by western blotting

Recently, we have reported multiple enzymes, such as urine diphosphate-galactose ceramide galactosyltransferase (UGT8), Aldo-keto reductase family 1 member B1 (AKR1B1) and phospholipid scramblase 1 (PLSCR1), were tightly associated with BLBC aggressiveness [14–16]. To search other possible metabolic molecules required for BLBC aggressiveness, we systematically analyzed gene expression profiles in multiple publicly available datasets, including TCGA, METABRIC, GSE25066, NKI295, GSE7390 and GSE22358, which contain over 4000 breast cancer patients [17–19]. Apart from some known genes that were previously identified to have key roles in BLBC, such as UGT8 and AKR1B1 [20]. AMD1 mRNA expression was much higher in BLBC than in other subtypes (Fig. 1A). To further investigate the correlation between AMD1 expression and basal subtype, we analyzed AMD1 mRNA expression in two gene expression datasets, E-TAMB-181 and E-TAMB-157, containing 56 and 51 breast cancer cell lines, respectively [21–24]. Similarly, high AMD1 expression was closely associated with basal subtype of breast cancer cell lines (Fig. 1B **and Figure ****S1**). Additionally, we affirmed these findings by qRT-PCR in different subtypes of breast cancer cell lines, showing that AMD1 mRNA expression was remarkably higher in BLBC cell lines (Fig. 1C **and** Fig. 1D). We further tested the protein levels of AMD1 in these cell lines. Consistently, AMD1 protein level also was higher in BLBC cell lines than in luminal cell lines (Fig. 1E). These findings indicate that AMD1 overexpression is positively associated with BLBC.

### AMD1 copy number amplification and hypomethylation of AMD1 promoter contribute to upregulated AMD1 expression in BLBC

**Fig. 2:**
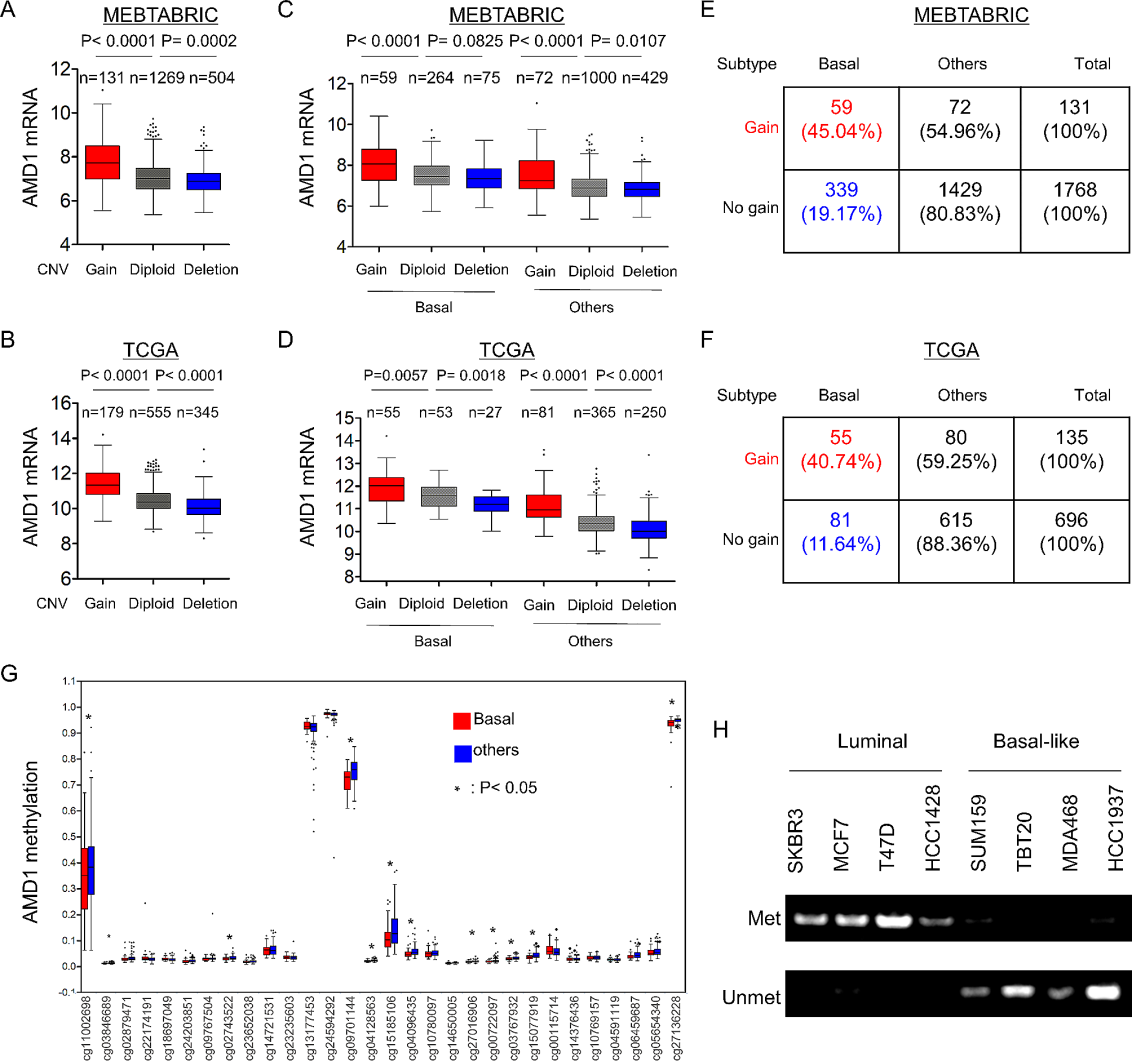
AMD1 overexpression correlated with its copy number amplification and promoter hypomethylation. (**A**, **B**) Box-plots indicated the correlation of AMD1 mRNA expression with its copy number variants status (gain, diploid and deletion) in breast cancer from MEBTABRIC dataset (**A**) and TCGA dataset (**B**). (**C**, **D**) Box-plots showed the association of AMD1 mRNA level with copy number variants (gain, diploid and deletion) in different subtypes of breast cancer from MEBTABRIC dataset (**C**) and TCGA dataset (**D**). (**E**, **F**) Analysis of the proportion of AMD1’s copy number status (gain or no gain) in different subtypes of breast cancer from MEBTABRIC dataset (**E**) and TCGA dataset (**F**). (**G**) Box-plots indicated AMD1 promoter methylation in different subtypes of breast cancer using multiple 450 K probes (TGCA dataset). (**H**) AMD1 promoter methylation was analyzed in breast cancer cell lines by Methylation-specific PCR.

Copy Number Variants (CNVs) are crucial components of genetic variations composed of deletion, duplication and other chromosomal changes, which closely predispose to human cancer [25]. To examine the effect of CNVs on AMD1 expression, we analyzed the copy number changes of AMD1 gene in METABRIC, TCGA and CCLE datasets. We observed that cases and cells with AMD1 amplification had much higher AMD1 expression than those with no amplification, supporting that AMD1 copy number amplification might correlate with high AMD1 level (Fig. 2A-B **and Figure ****S2****A**). We also analyzed the copy number changes in different subtypes of breast cancer tissues and cell lines, showing that AMD1 copy number amplification was predominantly correlated with BLBC subtype (Fig. 2C-F **and Figure ****S2****B-C**). These data strongly suggest that copy number amplification of AMD1 is tightly associated with AMD1 overexpression and BLBC subtype.

Aberrant DNA methylation is an critical regulator of gene transcription, being an epigenetic hallmark of many cancers [26]. To determine whether DNA methylation level was associated with AMD1expression in breast tumors, we analyzed methylation and expression of AMD1 from the TCGA dataset. The correlation between AMD1 expression examined by gene expression microarray and AMD1 methylation evaluated by 450 K Infinium microarray was analyzed, observing that the promoter regions of AMD1 in BLBC had a significant reduction in methylation compared with that in other subtypes (Fig. 2G). Notably, AMD1 mRNA expression was negatively correlated with AMD1 promoter methylation (**Figure ****S2****D**). In addition, Methylation-Specific PCR (MSP) analysis showed that the promoter regions of AMD1 in BLBC cell lines had remarkably less enrichment of methylation than that in luminal breast cancer cell lines (Fig. 2H). These data indicate that hypomethylation of AMD1 promoter is also important for AMD1 overexpression.

### AMD1 positively correlates with Sox10 and is a direct target of Sox10

**Fig. 3:**
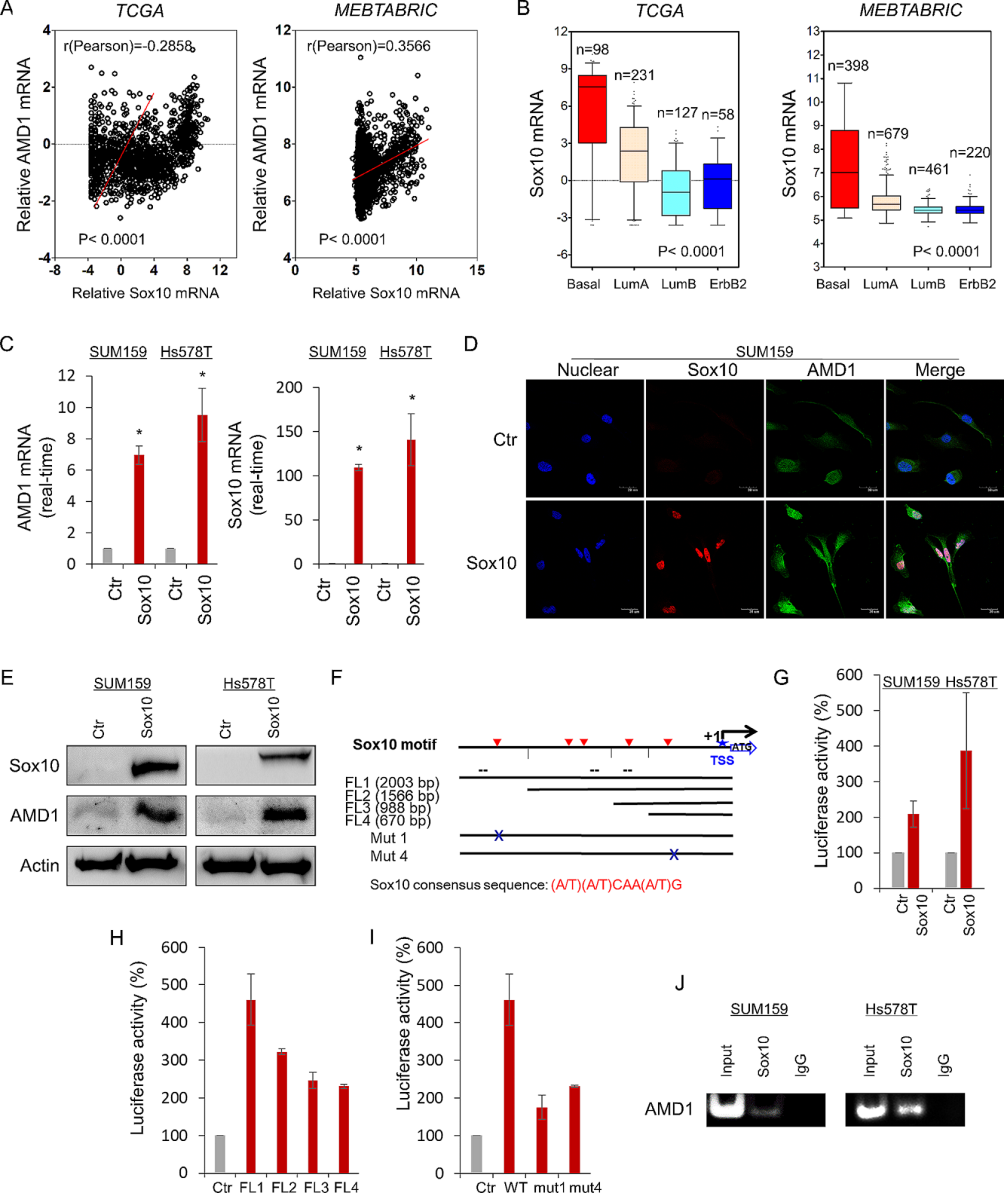
AMD1 positively correlates with Sox10 and is a direct transcriptional target of Sox10. (**A**) Analysis of TCGA and MEBTABRIC datasets for the expression of AMD1 and Sox10. The relative level of AMD1 was plotted against that of Sox10. (**B**) Box-plots indicated Sox10 mRNA expression in different subtypes of breast cancer from TCGA and MEBTABRIC datasets. (**C**) Expression of AMD1 and Sox10 was analyzed by quantitative real-time PCR in SUM159 and Hs578T cells infected with empty vector or Sox10-expressing vector. *p < 0.01 by Student’s t-test. (**D**) Expression of AMD1 and Sox10 was measured by immunofluorescent staining in SUM159 cells infected with empty vector or Sox10-expressing vector. Nuclei were visualized with DAPI (blue). Scale bar = 30 μm (right). (**E**) Expression of AMD1 and Sox10 was examined by western blotting in SUM159 and Hs578T cells infected with empty vector or Sox10-expressing vector. (**F**) Schematic diagram showed positions of potential Sox10-binding motifs in AMD1 promoter. AMD1 promoter luciferase construct and mutated derivatives were also displayed. Sox10 consensus sequence: (A/T)(A/T)CAA(A/T)G. (**G**) AMD1 promoter luciferase construct (FL1) was co-expressed with empty vector or Sox10-expressing vector in SUM159 and Hs578T cells, respectively. After 48 h, luciferase activities were analyzed (mean ± SD in three separate experiments). (**H**) AMD1 promoter luciferase constructs (FL1, FL2, FL3 and FL4) were co-expressed with empty vector or Sox10-expressing vector in HEK-293T cells. Luciferase activities were analyzed as in (**G**). (**I**) AMD1 promoter luciferase construct (FL1) as well as its mutants (mut1 and mut4) were co-expressed with empty vector or Sox10-expressing vector in HEK-293T cells. Luciferase activities were analyzed as in (**G**). (**H**) ChIP analysis for binding of Sox10 to the AMD1 promoter in SUM159 and Hs578T cells

Since many tumors with high expression of AMD1 did not have copy number amplification and hypomethylation of AMD1, other factors might associate with this event. Co-expression analysis of AMD1 with other genes in four datasets (TCGA, MEBTABRIC, NK295 and GSE25066) showed that AMD1 expression positively correlated with Sox10 expression (Fig. 3A **and Figure S3A**). We then analyzed Sox10 expression in different subtypes of breast cancer, observing that similar to AMD1, Sox10 was remarkably elevated in BLBC in four gene expression datasets (Fig. 3B **and Figure S3B**). To determine the causal relationship between AMD1 and Sox10, we expressed Sox10 in SUM159 and Hs578T cells. Remarkably, Sox10 upregulated AMD1 expression in mRNA and protein levels in both cell lines (Fig. 3C-E **and Figure S3C**). These results suggest that Sox10, as a transcriptional factor, may promote AMD1 expression through transcriptional activation.

Given their tight correlation between AMD1 and Sox10 and immediate induction of AMD1 expression by Sox10, we next investigate whether AMD1 expression was regulated directly by Sox10. AMD1 promoter contained five putative consensus Sox10-binding motifs (A/T)(A/T)CAA(A/T)G from − 2003 bp to transcription start site (TSS) (Fig. 3F). To test which locations and motifs are required for Sox10-mediated gene transcription, we cloned the human AMD1 promoter and generated several deletion mutants of promoter-luciferase constructs according to the location of these motifs, including FL1, FL2, FL3 and FL4 (Fig. 3F). By expressing the FL1 in SUM159 and Hs578T cells, an approximately two-to-four-fold increase in AMD1 promoter luciferase activity was observed in cells with Sox10 overexpression (Fig. 3G). Compared with the FL1, all the deletion mutants (FL2, FL3 and FL4) partially lost the reporter activity, whereas FL2 without region between − 2003 and − 1566 bp had much more reduction of the reporter activity to respond to Sox10 expression (FL2 vs. FL1), suggesting that the region between − 2003 and − 1566 bp is critical for Sox10-mediated AMD1 activation (Fig. 3H). FL4 without the region between − 2003 and − 670 bp did not further reduce the reporter activity compared with the FL3 without the region between − 2003 and − 988, whereas either FL3 or FL4 still remarkably enhanced the reporter activity compared with the control group without Sox10 expression, indicating that the region between − 670 and TSS is important for Sox10-mediated AMD1 activation (Fig. 3H). To further assess the binding motifs inside the AMD1 promoter, two constructs with point mutants were generated in the key Sox10-binding motifs (mut1 and mut4) **(**Fig. 3I**)**. Either the mut1 or the mut4 dramatically reduced the reporter activity induced by Sox10 **(**Fig. 3I**)**, indicating that Sox10 activates the AMD1 promoter in a Sox10 motif dependent fashion, and that the motifs in the regions between − 2003 and − 1566 bp, and between − 670 bp and TSS are required for Sox10-mediated transcriptional activation. To further determine whether Sox10 directly bound to the AMD1 promoter, we performed chromatin immunoprecipitation (ChIP) assays in SUM159 and Hs578T cells with Sox10 expression. A remarkable enrichment of Sox10 in the AMD1 promoter was observed in these cells (Fig. 3J). These data indicate that AMD1 is a direct target of Sox10.

**AMD1 expression activates the spermidine production and enhances breast cancer cell aggressiveness.**

**Fig. 4:**
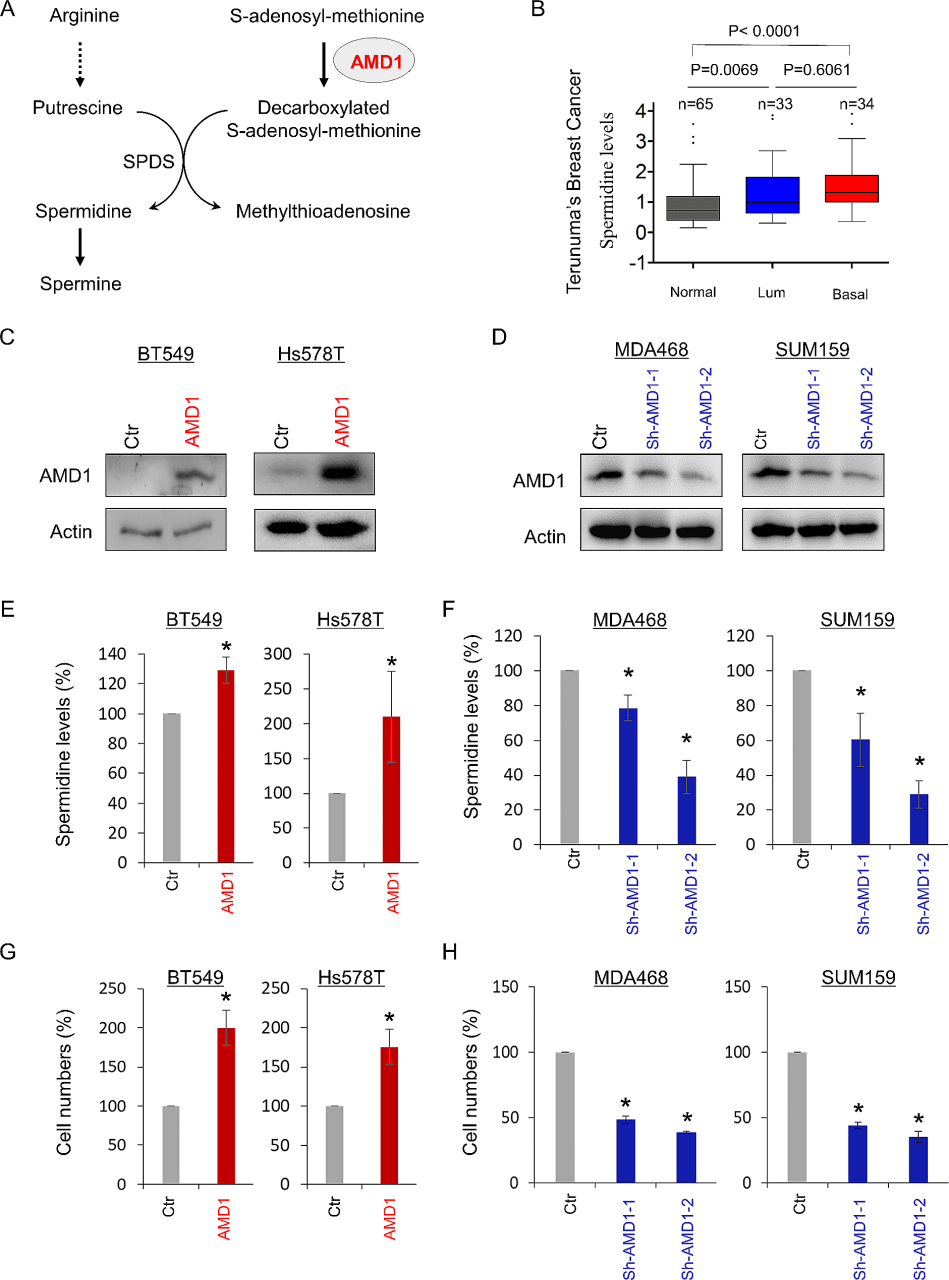
AMD1 promotes spermidine production and breast cancer cell proliferation. (**A**) Polyamine biosynthetic pathway. (**A**) Spermidine level was analyzed in normal breast tissue, luminal and BLBC tumors from Teunuma’s metabolomics dataset. (**C**, **D**) Stable clones with empty vector or AMD1 expression were also generated in BT549 and Hs578T cells (**C**), and stable transfectants with empty vector or knockdown of AMD1 expression were established in MDA-MB468 and SUM159 cells (**D**). (**E**, **F**) Level of spermidine was measured in BT549 and Hs578T cells with stable empty vector or ADM1 expression (**E**) as well as MB468 and SUM159 cells with stable empty vector or knockdown of AMD1 expression(**F**). (**G**, **H**) Growth of BT549 and Hs578T cells with stable empty vector or ADM1 expression (G) as well as MB468 and SUM159 cells with stable empty vector or knockdown of AMD1 expression (**H**) was measured by cell-count assay for 2 days. Data are shown as a percentage of control cells (mean ± SD in two independent experiments). *p < 0.01 by Student’s t-test

Spermidine is a key AMD1-mediated downstream metabolite in the polyamine biosynthetic pathway (Fig. 4A). We first analyzed the association of spermidine with breast cancer using the previous metabolomic data, showing that the spermidine level in both luminal and BLBC subtype, especially BLBC was significantly higher than that in normal breast tissue (Fig. 4B). To explore the association between spermidine and AMD1 expression, we created stable clones with empty vector or AMD1 expression in BT549 and Hs578T cells with low AMD1 expression, and also generated stable transfectants with empty vector or knockdown of AMD1 expression in MDA-MB468 and SUM159 cells with high AMD1 expression (Fig. 4C-D; **Figure S4A; and Figure ****S1**). We first examined the production of spermidine, showing that exogenous AMD1 expression caused a significant increase, whereas knockdown of AMD1 expression resulted in a dramatic decrease in spermidine levels (Fig. 4E-F), suggest that AMD1 is required for increased spermidine production in breast cancer cells. To further investigate the effect of AMD1 expression in tumor cell functions, we examined the effect of AMD1 expression on breast cancer cell proliferation, migration and invasion. AMD1 expression markedly induced the proliferation, migration and invasion of BT549 and Hs578T cells in vitro (Fig. 4G; and Figure S4, B and D). Consistently, knockdown of AMD1 expression dramatically repressed the proliferation, migration and invasion of MDA-MB468 and SUM159 cells in vitro (Fig. 4H; and Figure S4, C and E). These data indicate an important role of AMD1-mediated acquisition of breast cancer cell aggressiveness.

### AMD1-mediated eIF5A hypusination contributes to TCF4 protein translation

**Fig. 5:**
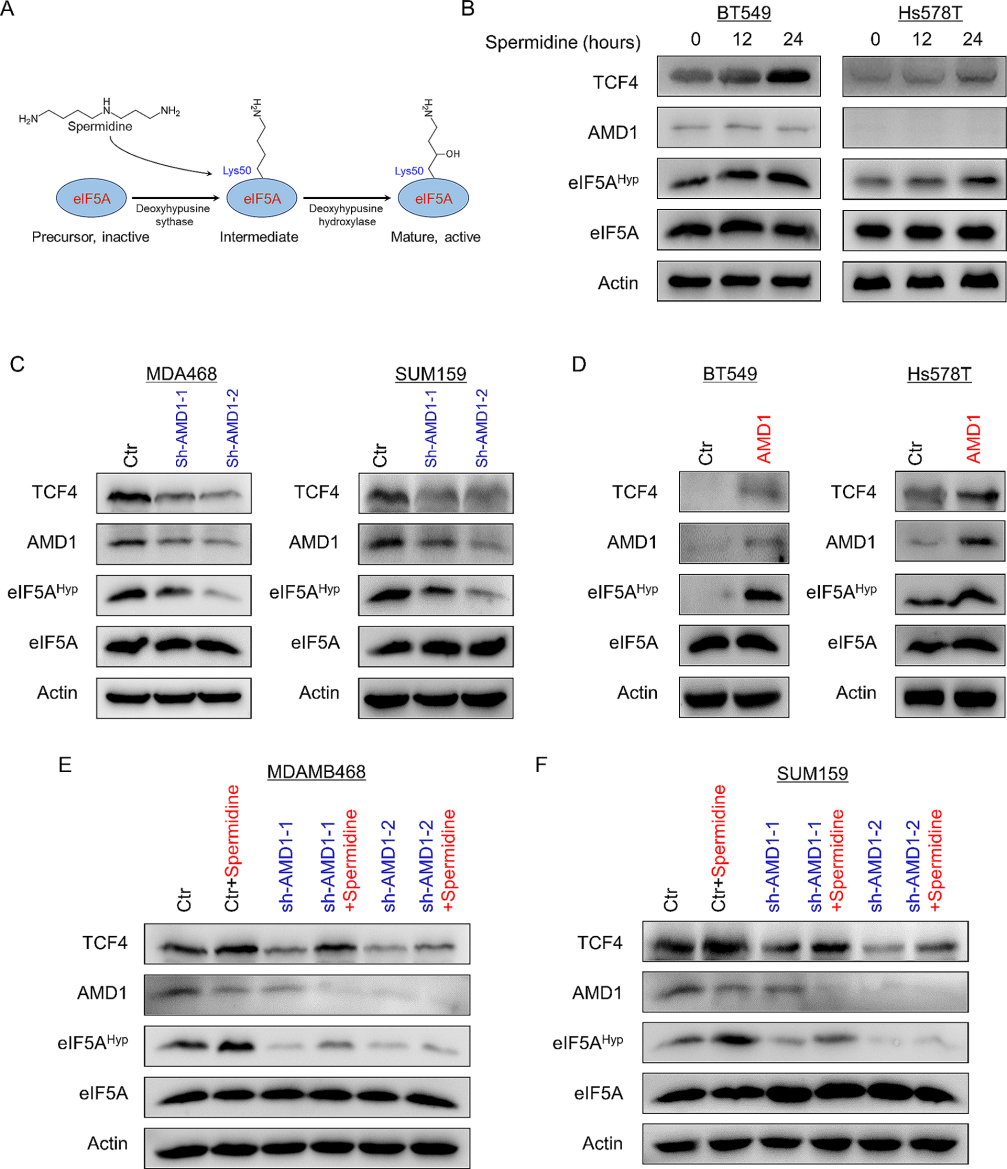
AMD1 increases eIF5A hypusination and TCF4 protein level. (**A**) Spermidine induces eIF5A hypusination that contributes to its activation. (**B**) Expression of TCF4, AMD1, eIF5A and eIF5A hypusination was examined by Western blotting in BT549 and Hs578T cells treated with or without spermidine (30ng/ml) for a period of 0, 12–24 h. (**C**, **D**) Expression of TCF4, AMD1, eIF5A and eIF5A hypusination was examined by Western blotting in MDA-MB468 and SUM159 cells with stable empty vector or knockdown of AMD1 expression (**C**) as well as BT549 and Hs578T cells with stable empty vector or ADM1 expression (**D**). (**E**, **F**) Expression of TCF4, AMD1, eIF5A and eIF5A hypusination was examined by Western blotting in MDA-MB468 (**E**) and SUM159 (**F**) cells with stable empty vector or knockdown of AMD1 expression following treatment with or without spermidine (30ng/ml) for a period of 24 h

AMD1 expression promoted spermidine production; contribution of spermidine to protein translation is to form an uncommon amino acid hypusine that is present in eIF5A to prevent ribosomal stalling during the translation of mRNAs encoding protein with polyproline (Fig. 5A). Indeed, spermidine treatment didn’t affect eIF5A level but markedly increased eIF5A hypusination in a time-dependent manner (Fig. 5B). In addition, we observed that spermidine treatment significantly promoted TCF4 level **(**Fig. 5B**)**. We also examined the effect of AMD1 expression in eIF5A hypusination and TCF4 level, showing that knockdown of AMD1 expression caused a dramatic decrease in eIF5A hypusination and TCF4 level of MDA-MB468 and SUM159 cells, whereas AMD1 expression resulted in a marked increase in eIF5A hypusination and TCF4 level of BT549 and Hs578T cells **(**Fig. 5C-D**)**. Additionally, we evaluated the effect of AMD1 expression or/and spermidine treatment in eIF5A hypusination and TCF4 level, observing that knockdown of AMD1 expression dramatically decreased, whereas spermidine significantly restored eIF5A hypusination and TCF4 level of MDA-MB468 and SUM159 cells **(**Fig. 5E-F**)**. These data suggest that AMD1-mediated spermidine production contributes to increased eIF5A hypusination and TCF4 level.

**Fig. 6:**
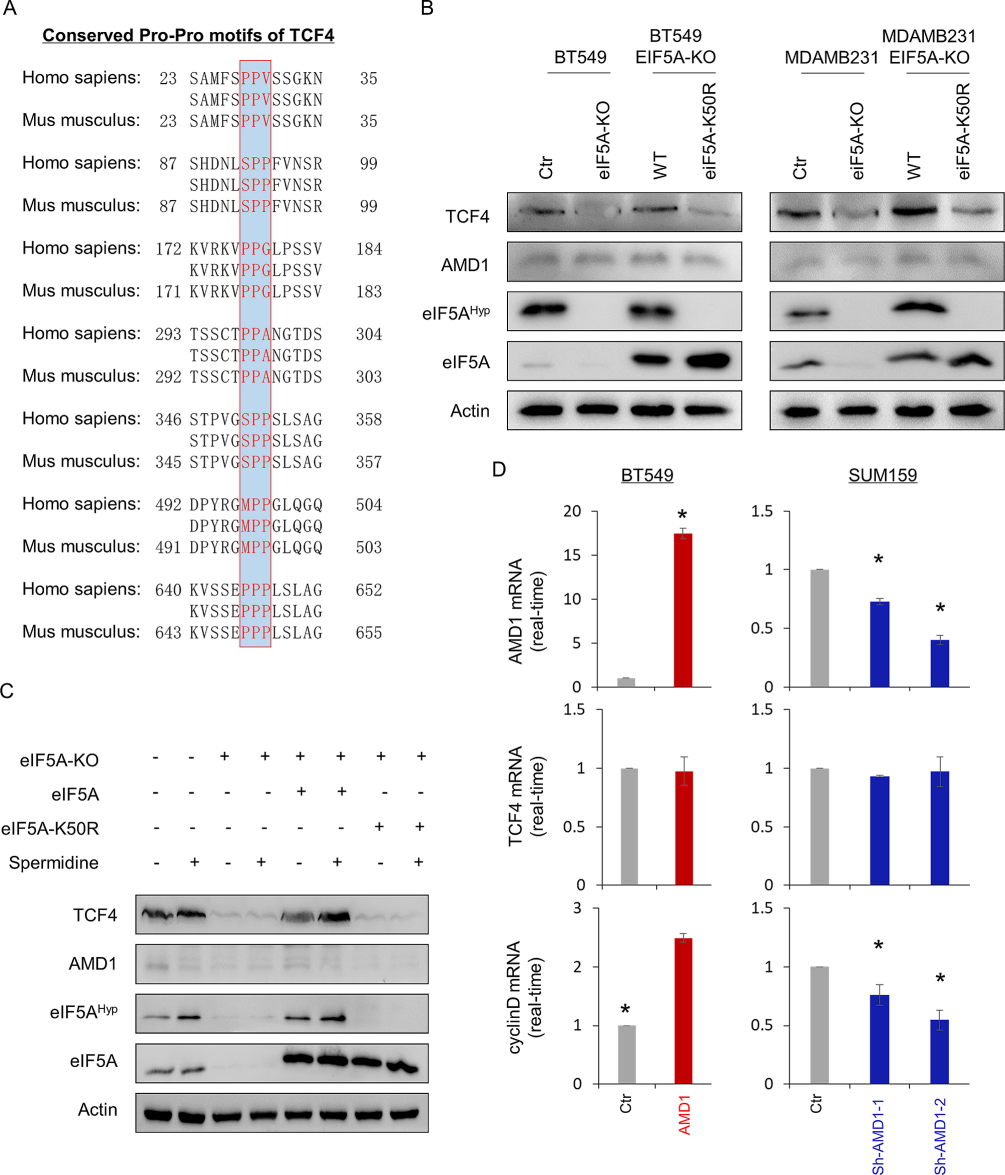
eIF5A hypusination contributes to TCF4 protein translation. (**A**) TCF4 contains 7 conserved Pro-Pro motifs. (**B**) Expression of TCF4, AMD1, eIF5A and eIF5A hypusination was examined by Western blotting in BT549 cells with or without eIF5A knockout (eIF5A-KO) as well as eIF5A-KO-expressing BT549 cells with eIF5A-WT or eIF5A-K50R expression. (**C**) Expression of TCF4, AMD1, eIF5A and eIF5A hypusination was examined by Western blotting in BT549 cells with or without eIF5A knockout (eIF5A-KO) as well as eIF5A-KO-expressing BT549 cells with eIF5A-WT or eIF5A-K50R expression following treatment with or without spermidine (30ng/ml) for a period of 24 h. (**D**) Expression of AMD1, TCF4 and cyclinD mRNA was analyzed by quantitative real-time PCR in BT549 cells with stable empty vector or ADM1 expression as well as SUM159 cells with stable empty vector or knockdown of AMD1 expression. Data are shown as mean ± SD based on three independent experiments

Active EIF5A alleviated ribosome pausing during translation of consecutive Pro-Pro motifs **(Figure S5A)** [27]. We analyzed the protein sequence of both human and mouse TCF4, observing the presence of seven potential Pro-Pro motifs conserved in both human and mouse (Fig. 6A). To assess the effect of eIF5A and its hypusination in TCF4 protein level, we generated stable clones, including knockout of eIF5A (eIF5A-KO), exogenous wild-type eIF5A (eIF5A-WT) or eIF5A mutant with K50R (eIF5A-K50R) that cannot be hypusinated in BT549 and MDA-MB231 cells. As expected, knockout of eIF5A lost eIF5A hypusination and markedly decreased TCF4 protein level, whereas re-expression of WT-eIF5A, but not eIF5A-K50R restored both eIF5A hypusination and TCF4 protein level (Fig. 6B). Additionally, we observed that spermidine treatment caused a significant increase in eIF5A hypusination and TCF4 protein level in BT549 cells with endogenous or exogenous eIF5A expression, but not in cells with eIF5A-KO or eIF5A-K50R (Fig. 6C). We also analyzed the effect of AMD1 expression in TCF4 and downstream target gene cyclinD mRNA expression, showing that AMD1 expression remarkably promoted, whereas knockdown of AMD1 significantly inhibited cyclinD mRNA expression; either AMD1 expression or knockdown of AMD1 expression didn’t affect TCF4 mRNA expression (Fig. 6D and **Figure S5B**). These data suggest that AMD1-mediated eIF5A hypusination promotes TCF4 protein level by enhancing translation of TCF4 with multiple Pro-Pro motifs.

### AMD1 promotes tumorigenicity of breast cancer cells

**Fig. 7:**
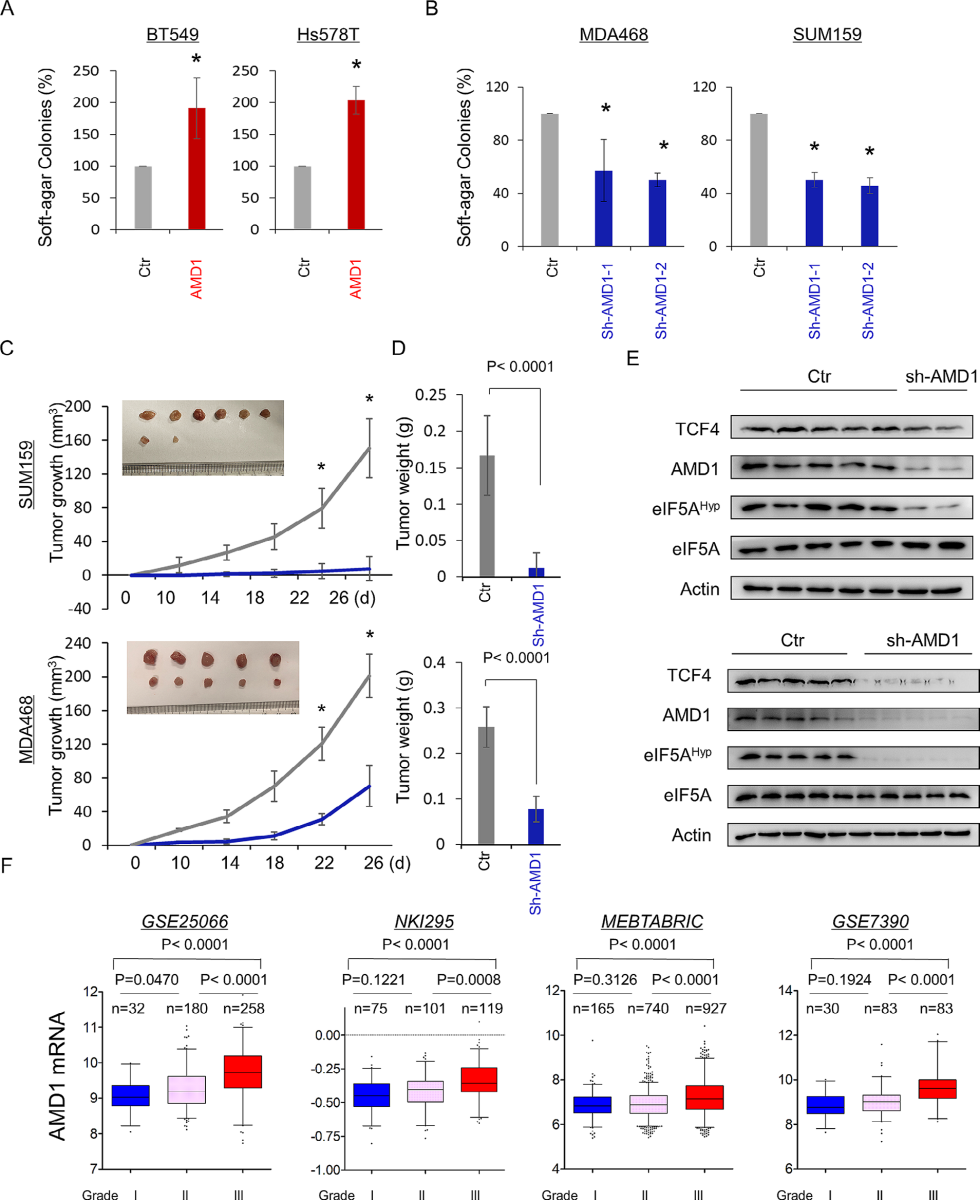
AMD1 promotes tumorigenicity in vitro and in vivo. (**A**, **B**) Soft-agar assay was performed using BT549 and Hs578T cells with or without AMD1 (**A**) as well as MDA-MB468 and SUM159 cells with stable empty vector or knockdown of AMD1 expression (**B**). Data are presented as the percentage of vector cell lines. Data are shown as mean ± SD based on three independent experiments. (**C**-**E**) MDA-MB468 and SUM159 cells with stable empty vector or knockdown of AMD1 expression were injected into the mammary fat pad of nude mice. Tumor growth was measured every four days. On day 26, mice were sacrificed and tumor weights were recorded. Tumor size (**C**) and weight (**D**) were measured and recorded. Expression of AMD1 was analyzed by Western blotting in tumor samples removed from two group mice (**E**). Data are presented as mean ± SEM of five mice. *p < 0.05 by Student’s t test. (**F**) Box-plots indicated AMD1 expression in different histological grades of breast cancer from GSE25066, NKI295, MEBTABRIC and GSE7390 datasets. Comparisons between two groups are made using the two-tailed Student’s t-test

**Fig. 8:**
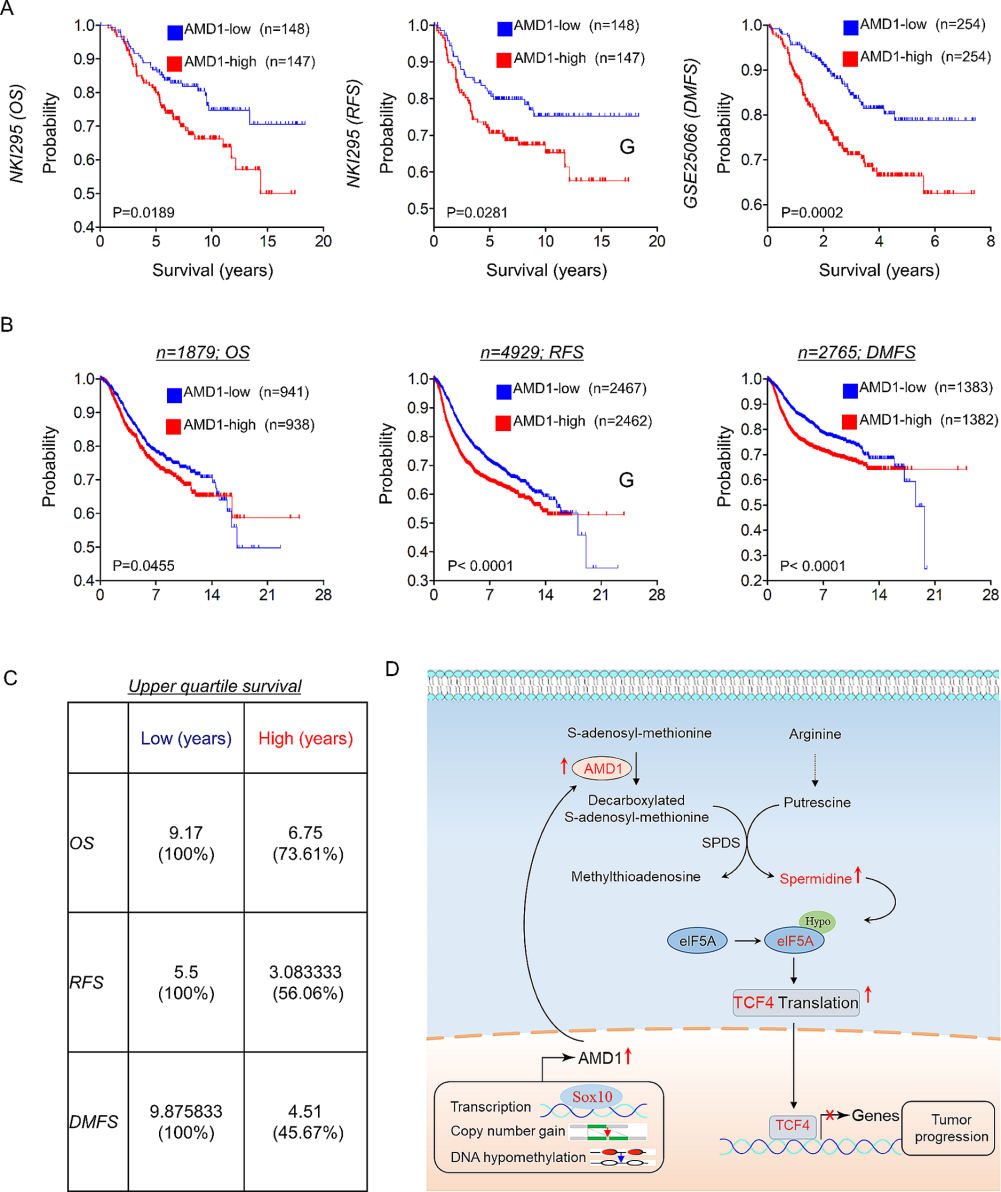
Elevated AMD1 predicts poor clinical outcome. (**A**) Kaplan-Meier survival analysis for OS, RFS, and DMFS of patients in NKI295 and GSE25066 datasets according to AMD1 expression status. The p value was determined using the log-rank test. (**B**) Kaplan-Meier survival analysis for OS, RFS, and DMFS of patients in an aggregate breast cancer dataset according to AMD1 expression status. The p value was determined using the log-rank test. (**C**) Analysis of the upper quartile survival for OS, RFS, and DMFS of patients in an aggregate breast cancer dataset. (**D**) A proposed model to illustrate mechanisms and functions of AMD1 overexpression and AMD1-mediated spermidine-eIF5A hypusination-TCF4 axis, promoting breast cancer aggressiveness (please see discussion)

Given the tight association of AMD1 with spermidine-eIF5A hypusination-TCF4 axis, we then evaluated the effect of AMD1 expression on the in vitro tumorigenicity using soft agar assay. AMD1 expression enhanced the ability of colony formation in BT549 and Hs578T cells, whereas knockdown of AMD1 significantly reduced of colonies in MDA-MB468 and SUM159 cells (Fig. 7A-B). Consistently, the CCK-8 cell viability assay showed that knockdown of AMD1 expression in MDA-MB468 and SUM159 cells was able to significantly inhibit cell proliferation (**Figure S6A**). Next, we examined tumorigenicity using xenograft models. Strikingly, MDA-MB468 and SUM159 cells with knockdown of AMD1 expression significantly inhibited tumor growth in vivo compared with wild-type control cells (Fig. 7C-D). We also examined AMD1 expression, TCF4 expression and eIF5A hypusination level in tumor samples from mouse model by Western blotting, showing that consistent with the analysis of cell lines, these tumor samples with knockdown of AMD1 expression had a dramatic decrease in AMD1 expression, TCF4 expression and eIF5A hypusination (Fig. 7E), indicating similar effects in vitro and in vivo. To further explore the clinical relevance of AMD1 expression in breast cancer progression, we assessed the correlation between AMD expression and histological grades of breast cancer patients. Using five datasets (GES25066, NKI295, MEBTABRIC, GES7390 and GES22358), in which patients were scored for the tumor grades, we observed that AMD1 was predominantly expressed in high tumor grade, especially in grade III (Fig. 7F **and Figure S6B**). We then sought to elucidate the association between AMD1 expression and patient survival in NKI295, GES25066 datasets and an aggregate breast cancer dataset by Kaplan-Meier survival analysis [17, 18, 28]. The analysis showed that patients with high expression of AMD1 had shorter overall survival (OS), relapse-free survival (RFS) and distant metastasis-free survival (DMFS) (Fig. 8A-B). The upper quartile survival analysis of the aggregate breast cancer dataset showed that patients with high expression of AMD1 had much lower survival than those with low expression of AMD1 (Fig. 8C). These clinical data strongly support the critical role of AMD1 in breast cancer aggressiveness.

## Discussion

In this study, we report that AMD1-mediated spermidine-eIF5A hypusination-TCF4 axis contributes to BLBC aggressiveness, providing new insights into the critical role of AMD1 in BLBC (Fig. 8D).

### AMD1 copy number amplification, hypomethylation of AMD1 promoter and transcription activation of Sox10 contribute to the overexpression of AMD1 in BLBC

CNVs are critical determinants for many cancers. Several CNVs associated with poor clinical outcomes have been reported in BLBC [25, 29, 30]. Our recent studies have shown that HIST1H1B or B3GNT3 overexpression is at least partially due to the copy number amplification [31, 32]. CNV is usually regarded as a key factor that associates with the changes of mRNA expression. Indeed, we observed that cases or cells with copy number amplification of AMD1 had much higher AMD1 expression than those without amplification by analyzing copy number variation in breast cancer tissues and cell lines from METABRIC, TCGA and CCLE datasets. It’s worth noting that cases and cells with AMD1 copy number amplification were positively associated with BLBC subtype. These data indicate that AMD1 copy number amplification is important for high AMD1 expression, especially in BLBC.

DNA hypermethylation leads to gene silencing [33], whereas DNA hypomethylation is closely corelated with gene transcription activation [34]. DNA hypomethylation causes transcription activation of oncogenes, regulating tumor-associated signaling pathway [35, 36]. Our data showed that low methylation level in promoter regions of AMD1 in breast cancer, especially in BLBC, positively correlated with high expression of AMD1 by analyzing AMD1 methylation and gene expression datasets. Together, these data indicate that the hypomethylation of AMD1 promoter is another important mediator for high AMD1 expression.

Some breast tumors with AMD1 overexpression were observed to have no AMD1 copy number amplification and hypomethylation of AMD1 promoter, indicating the involvement of other mediators in the upregulation of AMD1 expression. Sox10, a transcriptional activator is highly expressed in BLBC, which is critical for tumor progression [37–39]. Our data showed that Sox10 had a positive correlation with AMD1, and bound to AMD1’s promoter with multiple putative consensus Sox10-binding motifs to induce AMD1 transcription, supporting that Sox10 is a direct transcriptional activator responsible for high AMD1 expression in BLBC.

### AMD1-mediated spermidine production activates eIF5A hypusination-TCF4 axis

Polyamines are essential for normal cell growth; however, polyamine metabolism is frequently dysregulated in cancer. It has been reported that elevated polyamine levels are required for transformation and tumor progression [40, 41]. Spermidine is an important AMD1-mediated polyamine [1]. Our data showed that AMD1 significantly induced spermidine production in breast cancer cells, and the spermidine level in BLBC subtype with high AMD1 expression was much higher than that in normal breast tissue and luminal subtype. Spermidine serves as a substrate for the hypusination of eIF5A by two enzymes, deoxyhypusine synthase (DHPS) and deoxyhypusine hydroxylase (DOHH) [1]. Hypusinated eIF5A has a critical role in activating eIF5A to promote protein translation elongation by relieving translational pausing of ribosomes at specific stalling motifs (Pro-Pro motif) [27]. Our dada showed that AMD1-mediated spermidine production markedly upregulated eIF5A hypusination. So far, only a few target genes associated with eIF5A hypusination have been reported in tumor cells, such RhoA and MYC [4, 5]. Given the critical roles of AMD1-mediated eIF5A hypusination in BLBC, we checked the molecules in Wnt/TCF signaling, TGF-β and NF-κB pathways that are involved in BLBC progression [9, 42–44]. TCF4, a key transcription factor is involved in the Wnt signaling pathway [7, 8]. In breast cancer, activating Wnt/TCF signaling contributes to breast cancer, especially BLBC progression and drug resistance [9]. Our data showed that TCF4 contained seven conserved Pro-Pro motifs, and AMD1 expression or spermidine treatment significantly enhanced eIF5A hypusination and TCF4 protein level in cells with endogenous or exogenous eIF5A expression, but not in cells with eIF5A-KO or eIF5A-K50R. These data strongly support that AMD1-mediated spermidine production and subsequent eIF5A hypusination upregulates TCF4 protein level by promoting translation of TCF4 with multiple conserved Pro-Pro motifs.

### AMD1-mediated spermidine-eIF5A hypusination-TCF4 axis represents a potential prognostic indicator and therapeutic targets for BLBC

Given the tight association of AMD1 with breast cancer, it was important to assess whether AMD1 is appropriate as a prognostic factor for breast cancer patients. Several factors that may predict patient prognosis, have been identified, including (1) Breast cancer subtypes: AMD1 expression is especially upregulated in BLBC; (2) Tumor grade: high AMD1 expression is correlated with higher tumor grade; (4) tumor metastasis: high AMD1 expression has a much higher probability of distant metastasis and metastatic dissemination to the brain and lungs that is line with the metastatic propensity of BLBC; (3) Survival rate: high AMD1 expression predicts poorer survival in breast cancer patients. These findings support AMD1 as a promising prognostic biomarker for breast cancer patients.

Our studies showed that AMD1-mediated spermidine biosynthetic pathway in BLBC cells contributed to tumor cell proliferation and tumor growth. Clinically, high AMD1 expression occurs specifically in BLBC and predicts poor prognosis. Clearly, our study indicates that AMD1-mediated spermidine-eIF5A hypusination-TCF4 axis represents an oncogenic event responsible for BLBC aggressiveness. TCF4, as a transcription factor, is difficult to target therapeutically due to lack of a clear ligand-binding domain. As upstream targets in AMD1-mediated metabolic system of polyamine, AMD1 and enzymes associated with eIF5A hypusination might be potentially valuable for therapeutics against BLBC. Notably, several inhibitors of AMD1 and eIF5A hypusination are available [1]; however most of them have failed for clinical use due to unmanageable toxicity. Further elucidating AMD1-mediated spermidine-eIF5A hypusination-TCF4 axis and developing corresponding antagonistic drugs may improve our prospects for developing effective prevention and treatment strategies against BLBC.

## Conclusions

To summarize, we demonstrate that AMD1 activates the spermidine-eIF5A hypusination-TCF4 axis, offering a solid link between AMD1-mediated spermidine-eIF5A hypusination-TCF4 axis and BLBC aggressiveness, indicating potential prognostic indicators and therapeutic targets for BLBC.

### Electronic supplementary material

Below is the link to the electronic supplementary material.

Supplementary Material 1

Supplementary Material 2

## Data Availability

No datasets were generated or analysed during the current study.

## Abbreviations

AMD1: S-adenosylmethionine decarboxylase proenzyme
eIF5A: eukaryotic initiation factor 5 A isoform 1
TCF4: transcription factor 4
BLBC: basal-like breast cancer
CNVs: copy number variants
RT-qPCR: real-time quantitative polymerase chain reaction
DMFS: distant metastasis-free survival
OS: overall survival
RFS: relapse-free survival
