# Supplementary material for: AMD1 promotes breast cancer aggressiveness via a spermidine-eIF5A hypusination-TCF4 axis
Source: Breast Cancer Res. 2024 Apr 23;26:70. doi: 10.1186/s13058-024-01825-6 (PMC11040792; doi:10.1186/s13058-024-01825-6)
Supplement: Supplementary file 1 — Supplementary Material 1 [file 13058_2024_1825_MOESM1_ESM.doc]

**SUPPLEMENTARY FIGURE LEGENDS**

**
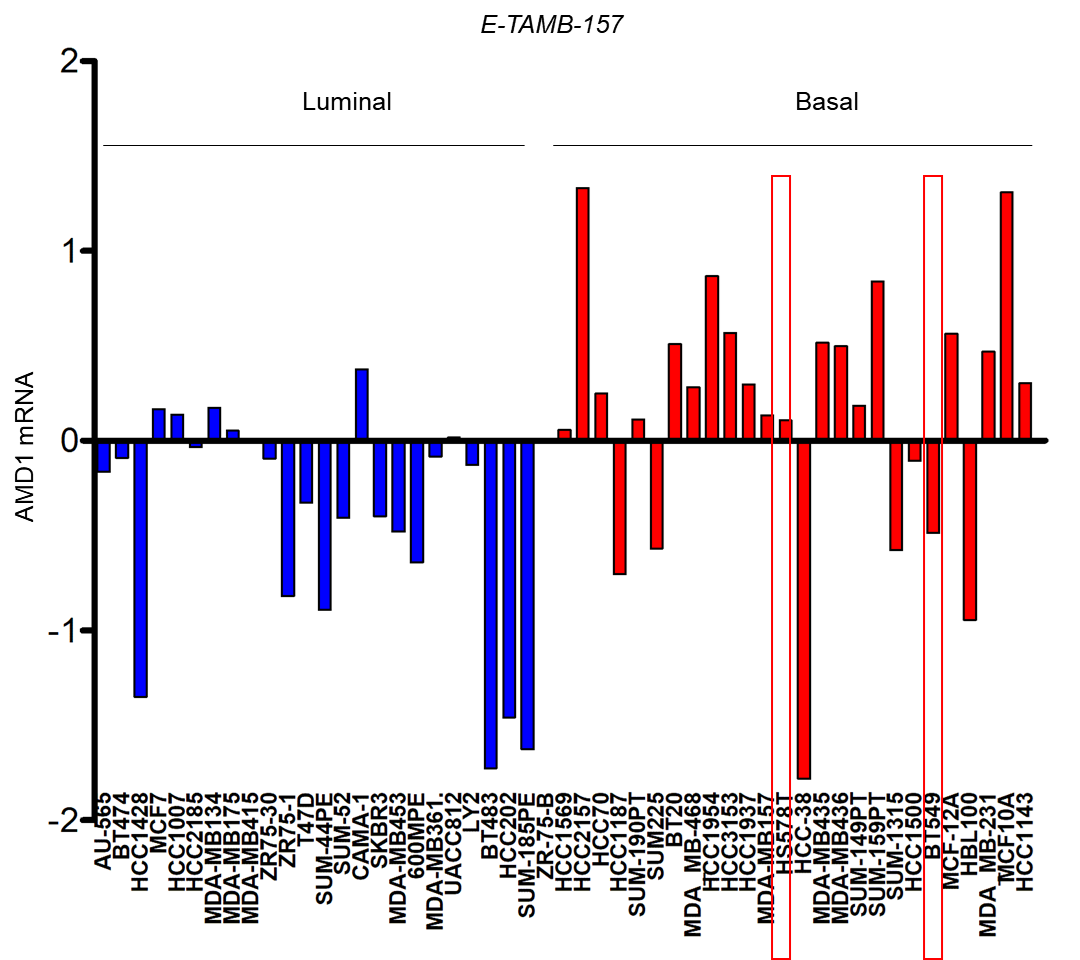
**

**Figure S1. AMD1 mRNA expression was shown in BLBC and luminal cell lines from the E-TAMB-157 dataset.**

**
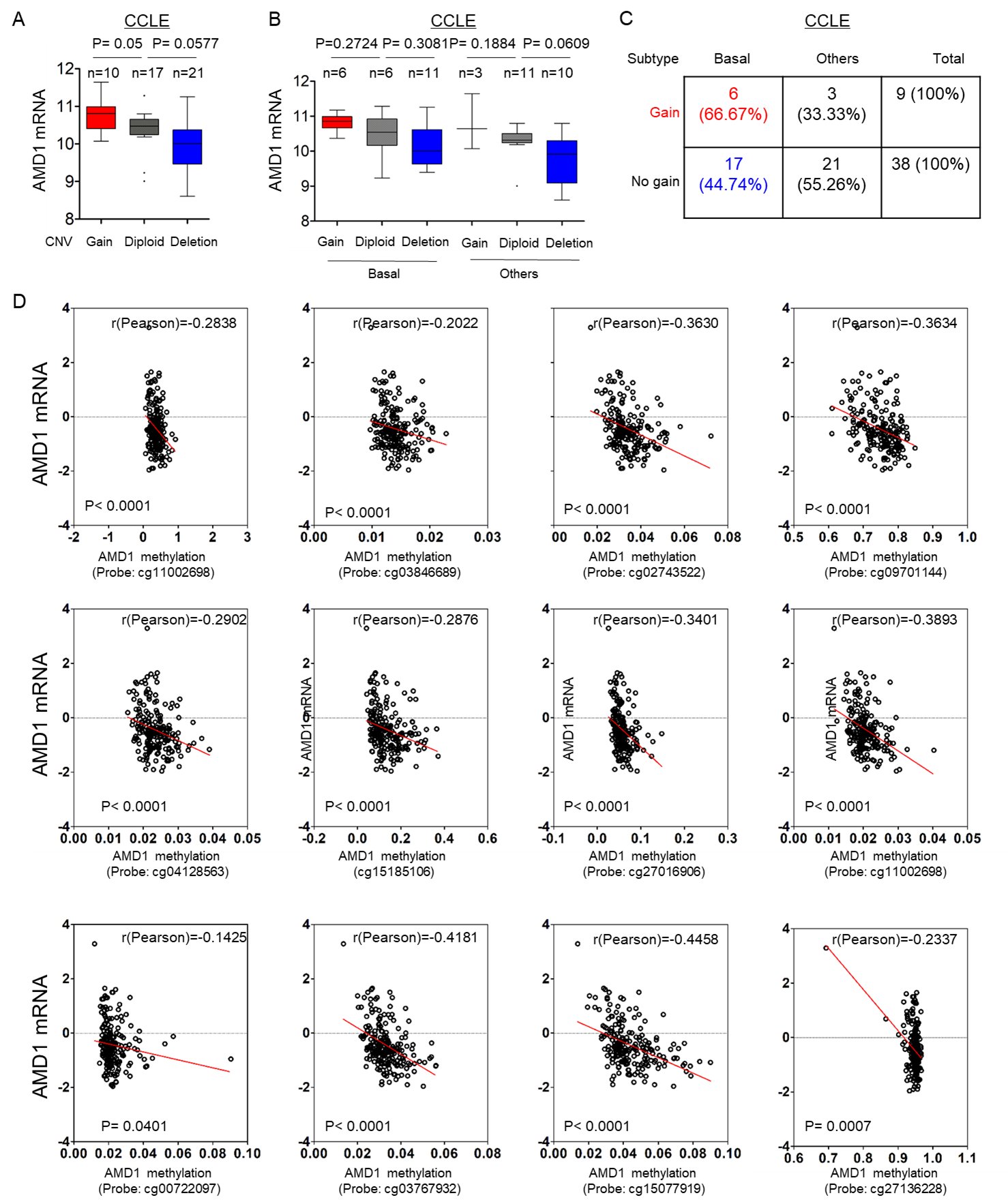
**

**Figure S2. AMD1 overexpression correlated with its copy number amplification and promoter hypomethylation.**

(A) Box-plots indicated the correlation of AMD1 mRNA expression with its copy number variants status (gain, diploid and deletion) in breast cancer from the CCLE dataset.

(B) Box-plots showed the association of AMD1 mRNA level with copy number variants (gain, diploid and deletion) in different subtypes of breast cancer from the CCLE dataset.

(C) Analysis of the proportion of AMD1’s copy number status (gain or no gain) in in different subtypes of breast cancer from the CCLE dataset.

(D) Analysis of the correlation between AMD1 mRNA expression and its copy number status (gain or no gain) in breast cancer from the TCGA dataset using multiple 450 K probes (TGCA dataset).

**
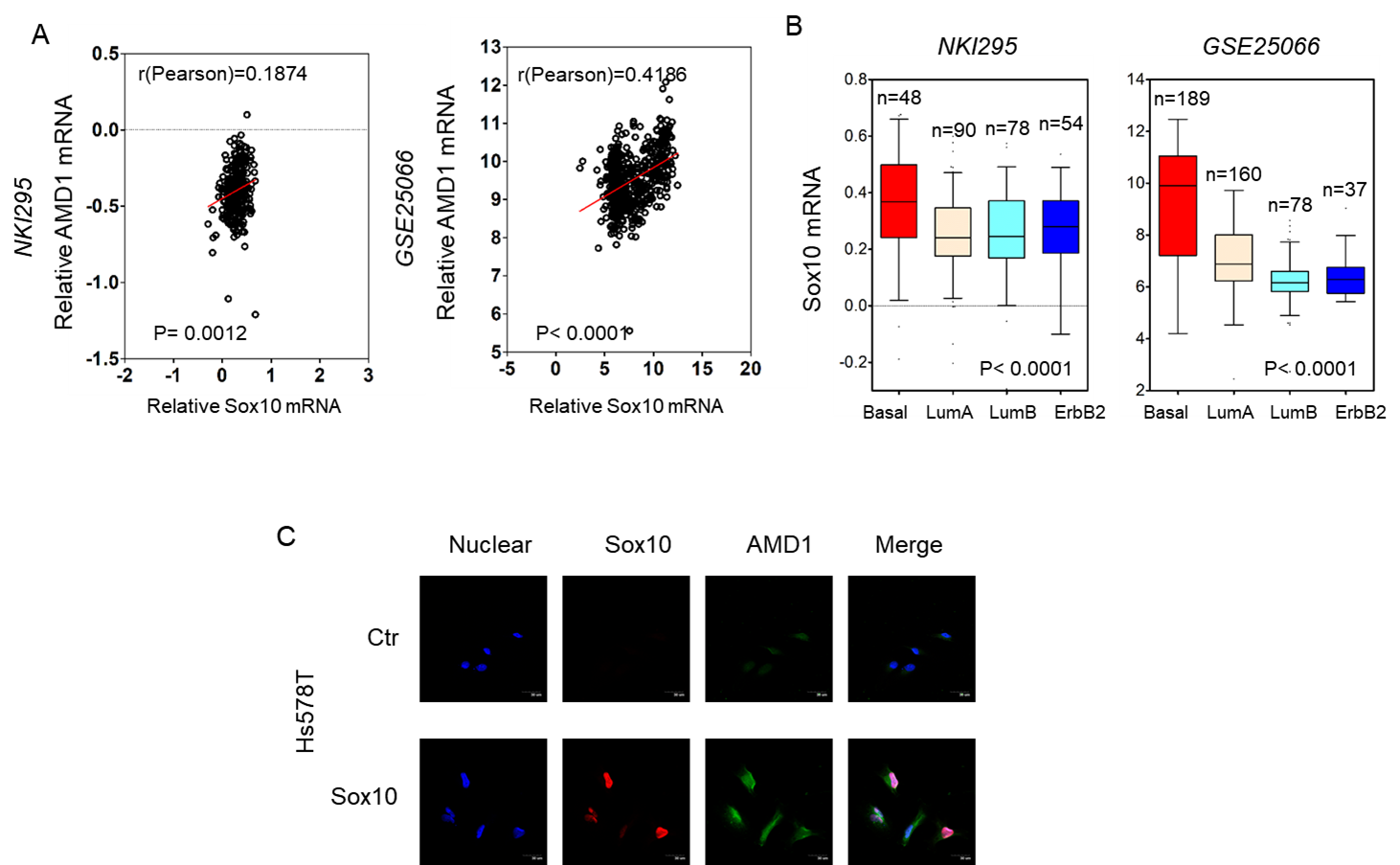
**

**Figure S3. AMD1 positively correlates with** **Sox10**.

(A) Analysis of NKI295 and GSE25066 datasets for the expression of AMD1 and Sox10. The relative level of AMD1 was plotted against that of Sox10.

(B) Box-plots indicated Sox10 mRNA expression in different subtypes of breast cancer from NKI295 and GSE25066 datasets.

(C) Expression of AMD1 and Sox10 was measured by immunofluorescent staining in Hs578T cells infected with empty vector or Sox10-expressing vector. Nuclei were visualized with DAPI (blue). Scale bar = 30 μm (right).

**
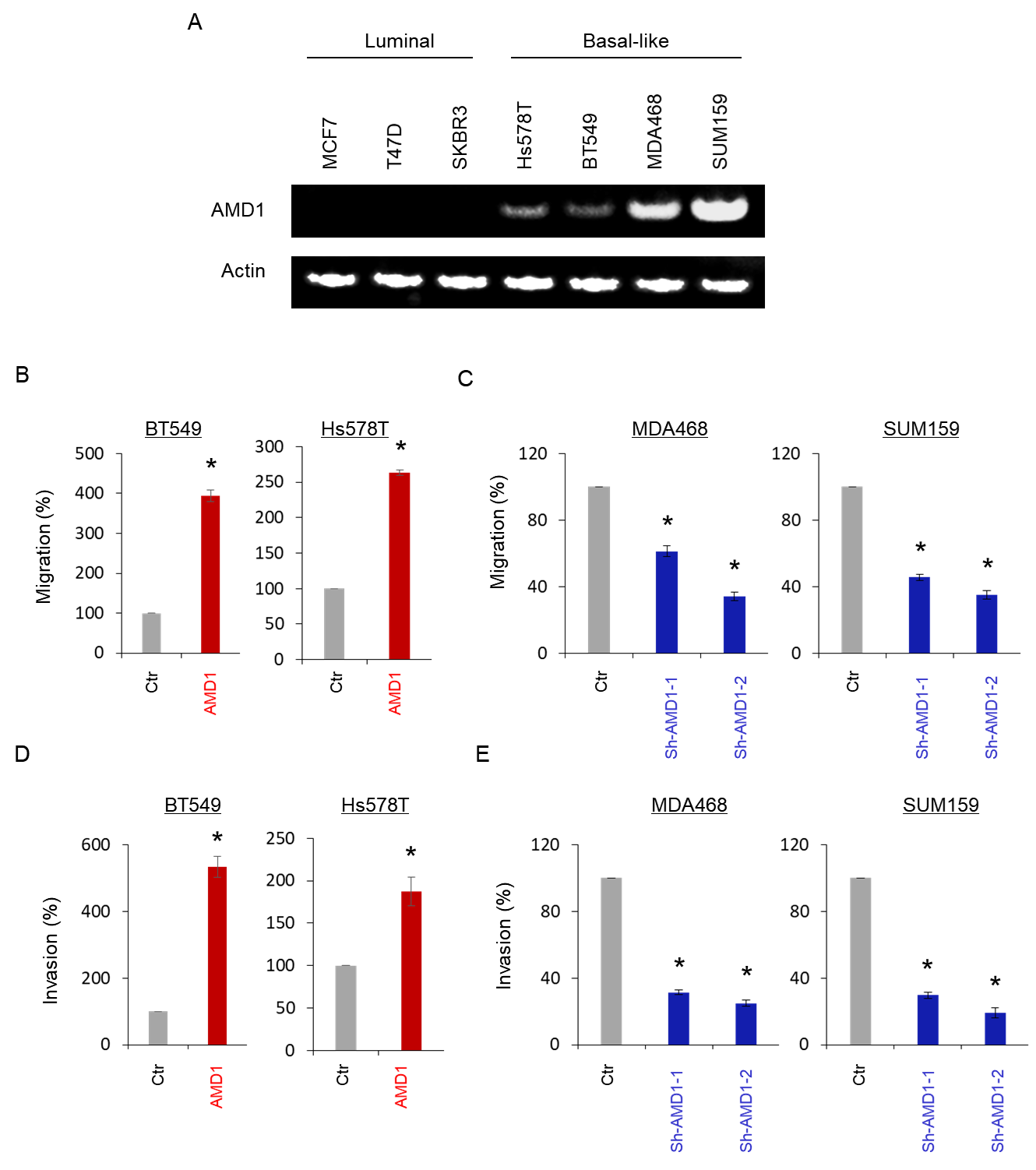
**

**Figure S4. AMD1 enhances breast cancer cell migration and invasion.**

(A) Expression of AMD1 mRNA was analyzed by semiquantitative RT-PCR in breast cancer cell lines.

(B-E) Migratory ability (B, C) and invasiveness (D, E) of BT549 and Hs578T cells with stable empty vector or ADM1 expression (B, D) as well as MB468 and SUM159 cells with stable empty vector or knockdown of AMD1 expression (C, E) was measured. The percentage of migratory and invasive cells was shown in the bar graph (mean ± SD in three separate experiments). *p< 0.01 by Student’s t-test.


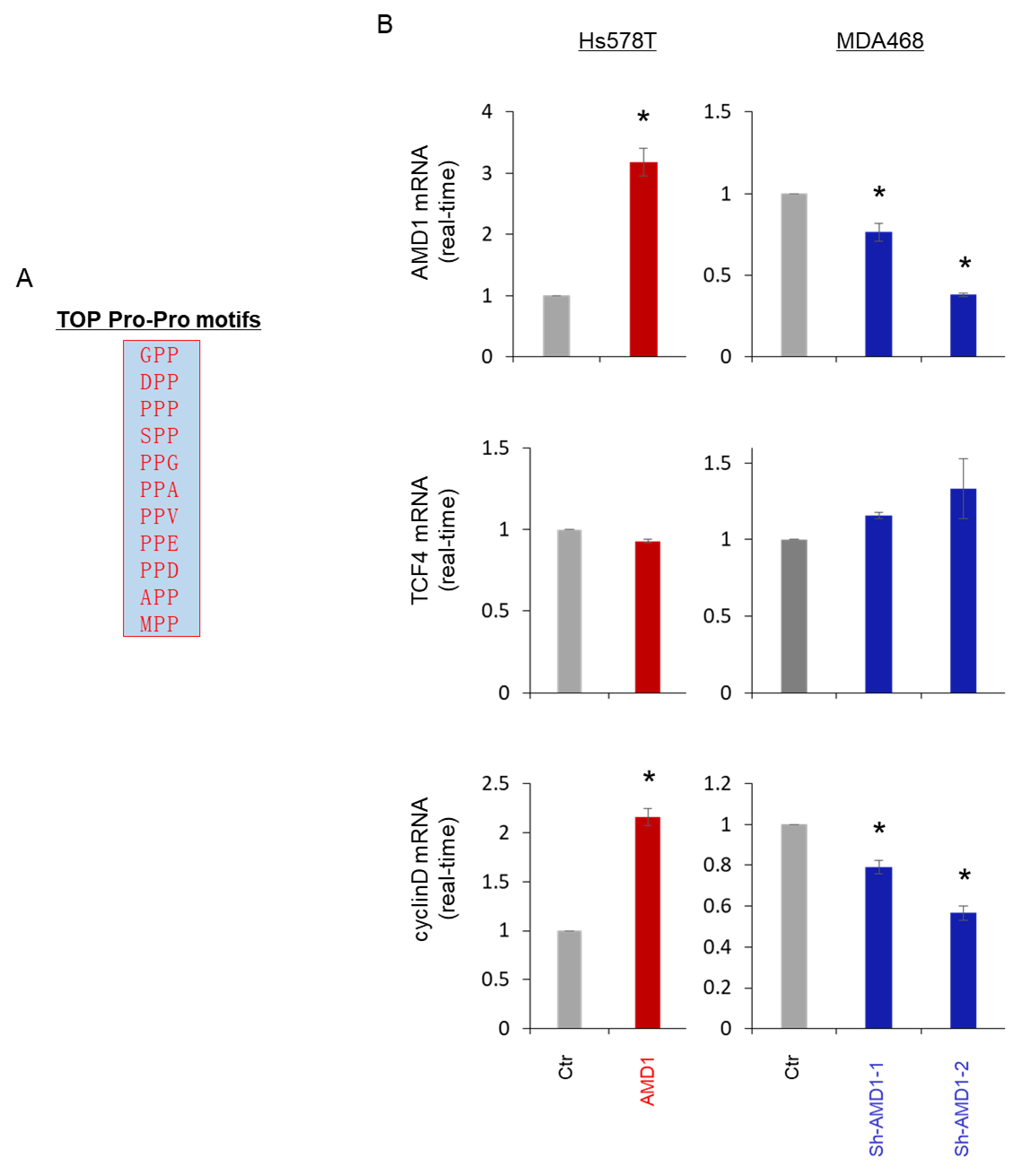


**Figure S5.**  **eIF5A hypusination contributes to TCF4 protein translation**

(A) Top conserved Pro-Pro motifs.

(B) Expression of AMD1, TCF4 and cyclinD mRNA was analyzed by quantitative real-time PCR in Hs578T cells with stable empty vector or AMD1 expression (left panel) as well as MDA-MB468 cells with stable empty vector or knockdown of AMD1 expression (right panel). Data are shown as mean ± SD based on three independent experiments.


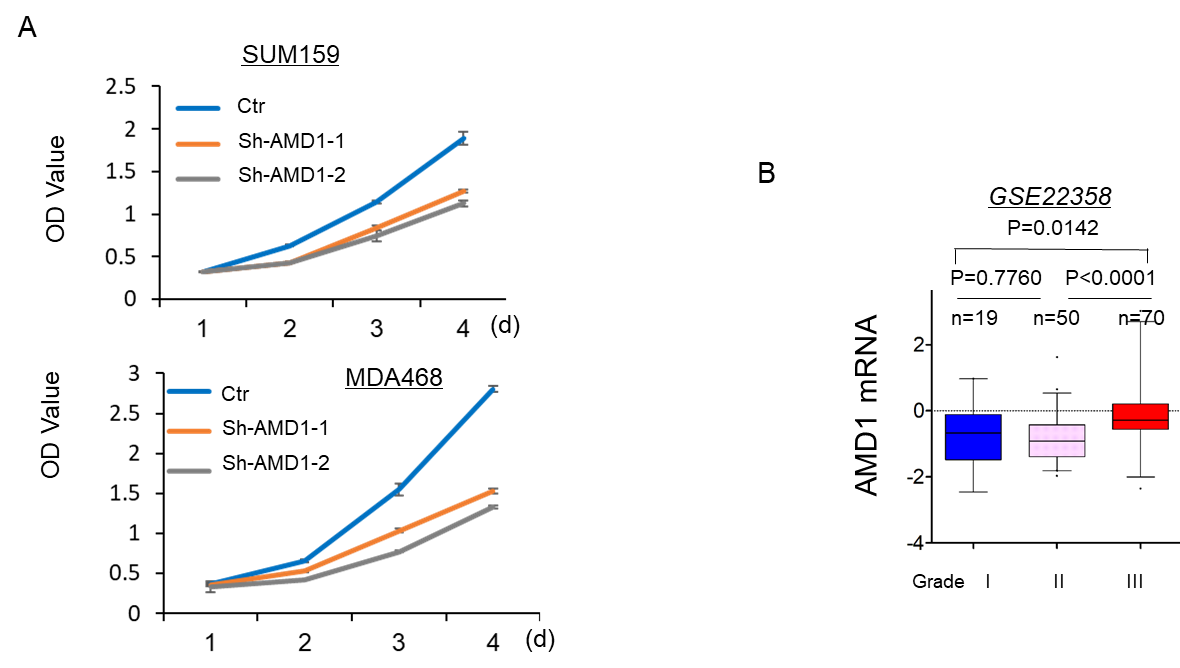


**Figure S6. Elevated AMD1 correlates with higher histological grades of breast cancer.**

(A) CCK-8 assay was performed using MDA-MB468 and SUM159 cells with stable empty vector or knockdown of AMD1 expression .

(B)Box-plots indicated AMD1 expression in different histological grades of breast cancer from GSE22358 dataset. Comparisons between two groups are made using the two-tailed Student's t-test.
