# Supplementary material for: AMD1 promotes breast cancer aggressiveness via a spermidine-eIF5A hypusination-TCF4 axis
Source: Breast Cancer Res. 2024 Apr 23;26:70. doi: 10.1186/s13058-024-01825-6 (PMC11040792; doi:10.1186/s13058-024-01825-6)
Supplement: Supplementary file 2 — Supplementary Material 2 [file 13058_2024_1825_MOESM2_ESM.pdf]

Figure 1E

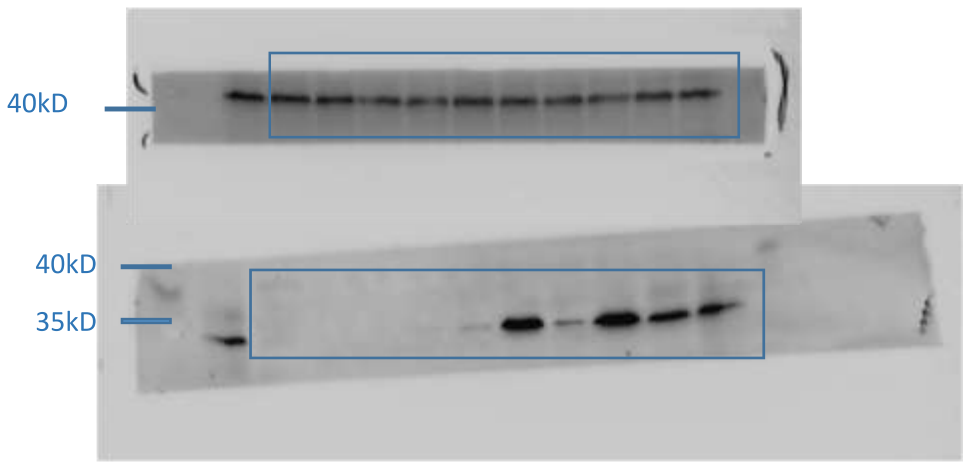

Figure 3E

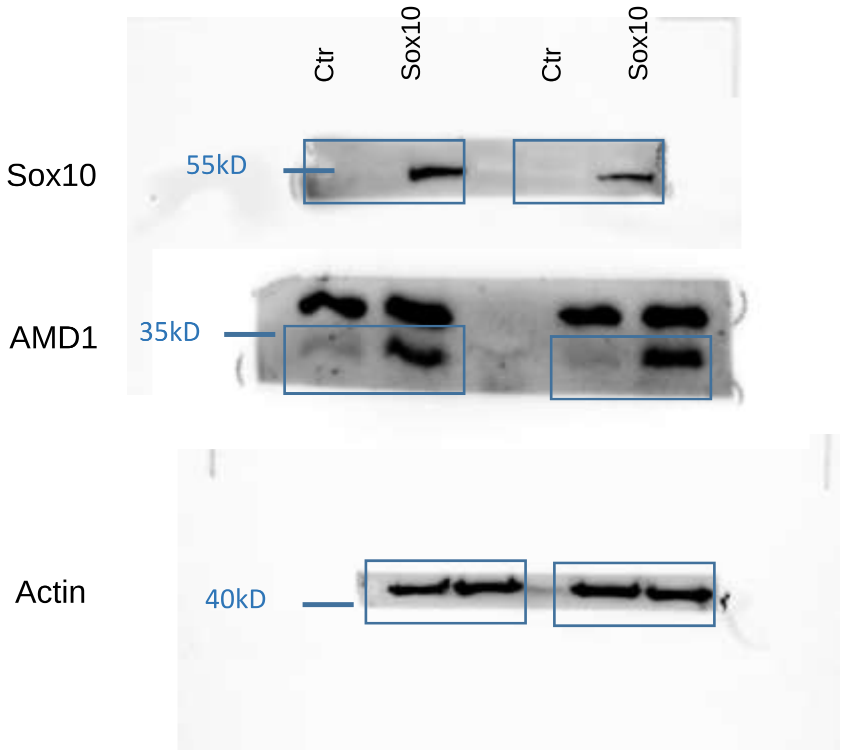

Figure 4C

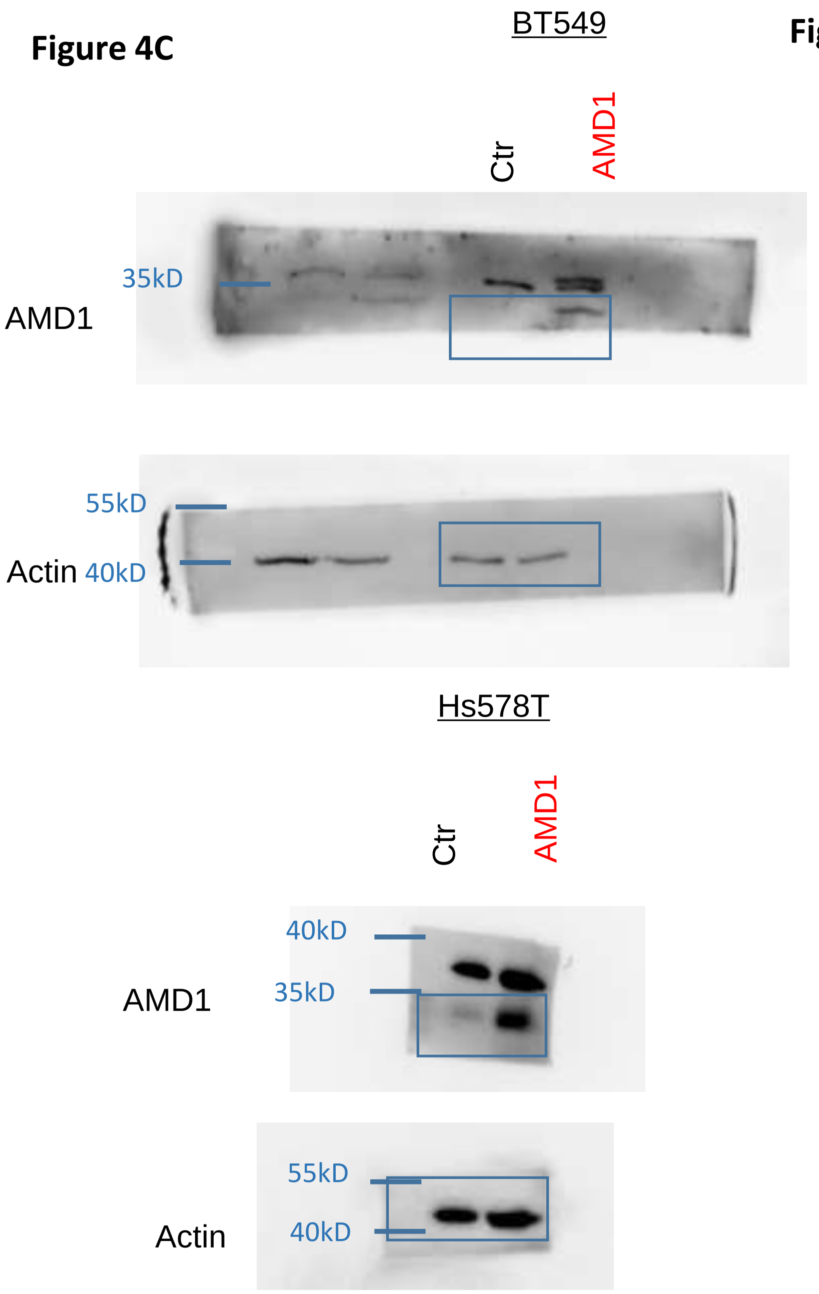

Figure 4D

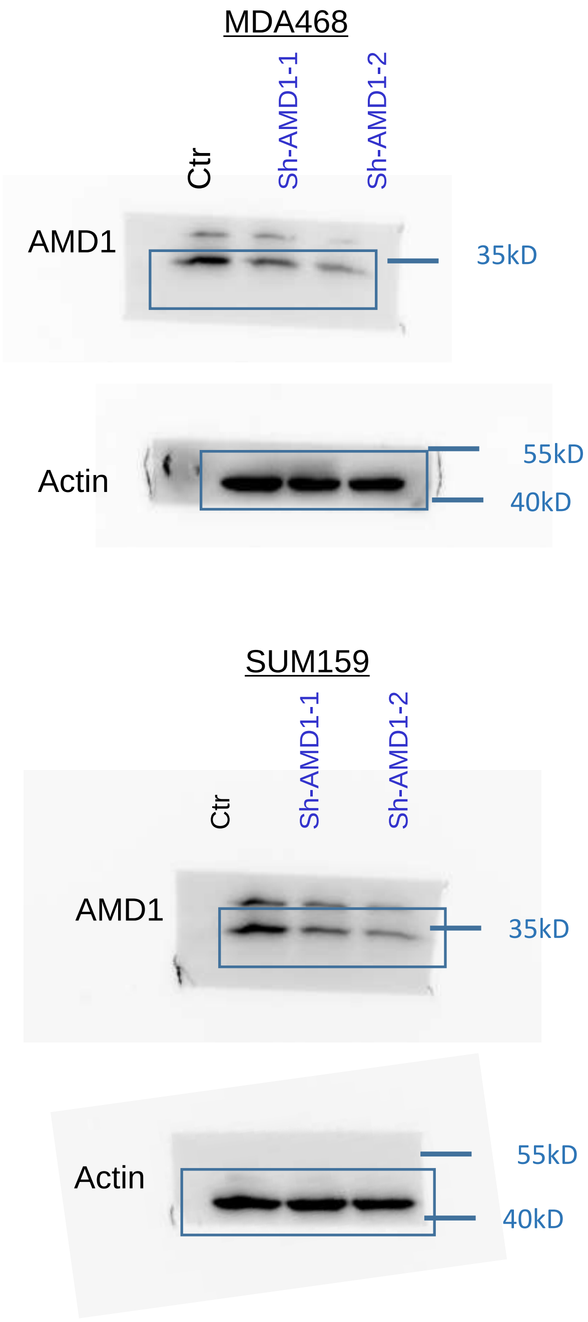

Figure 5B

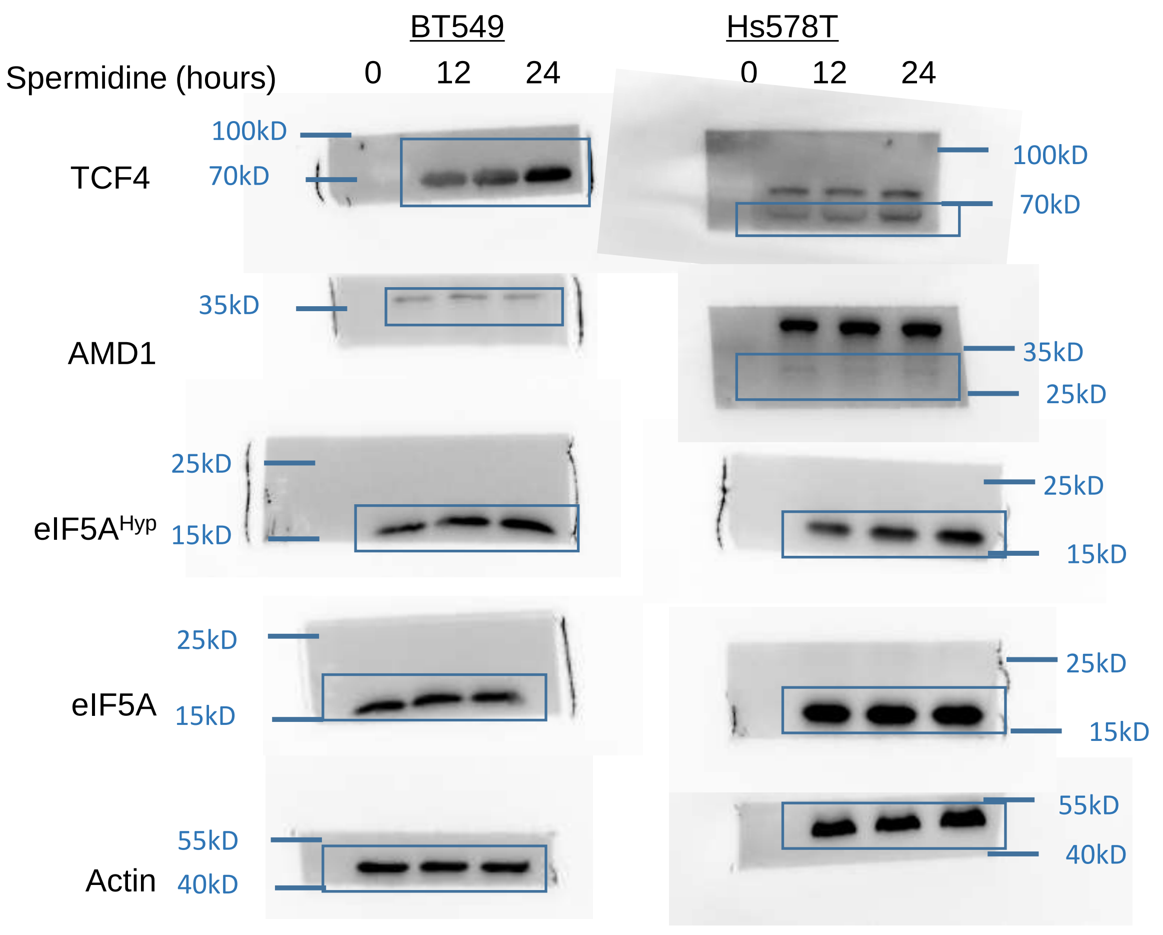

Figure 2H

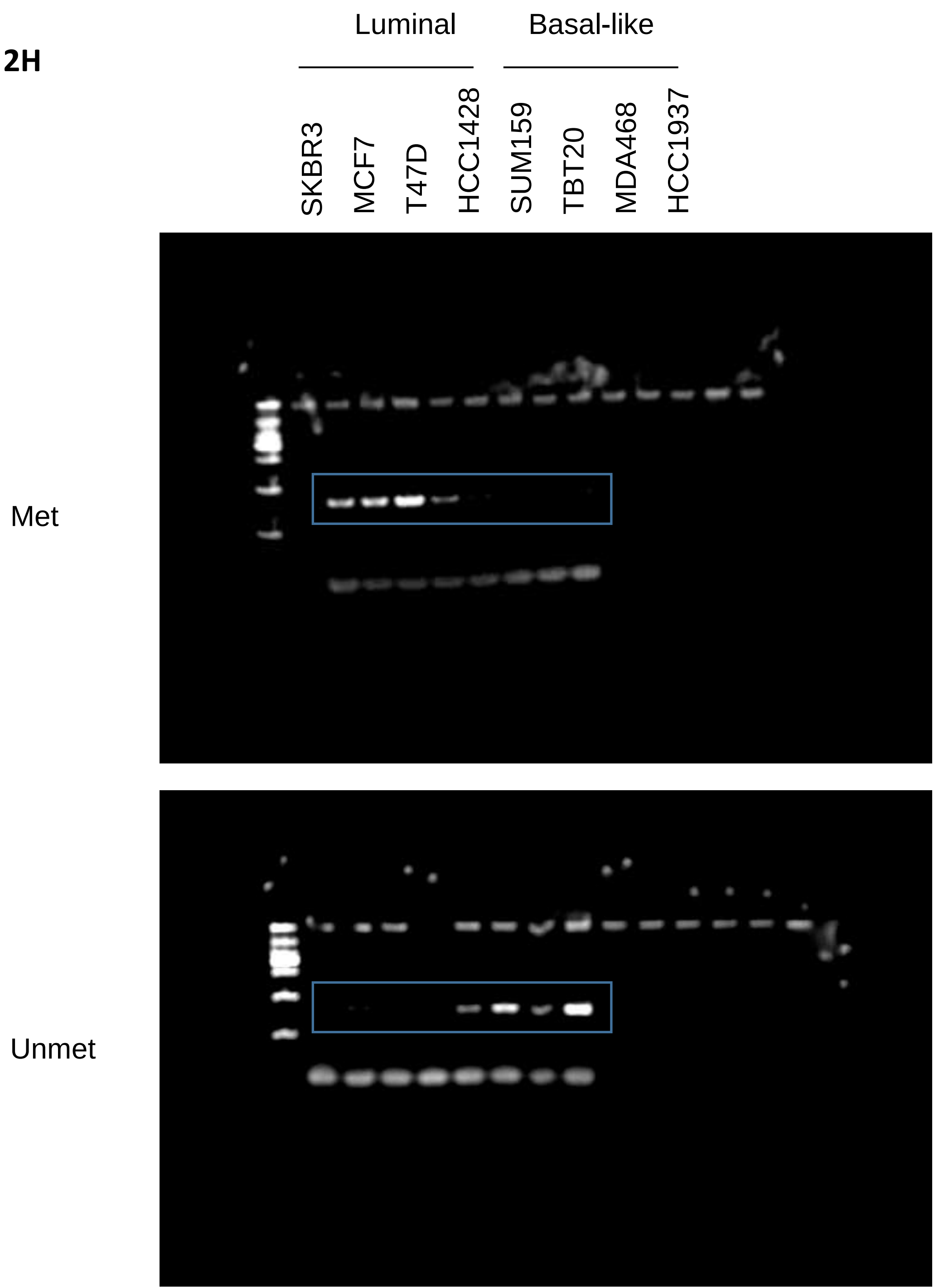

Figure 5C

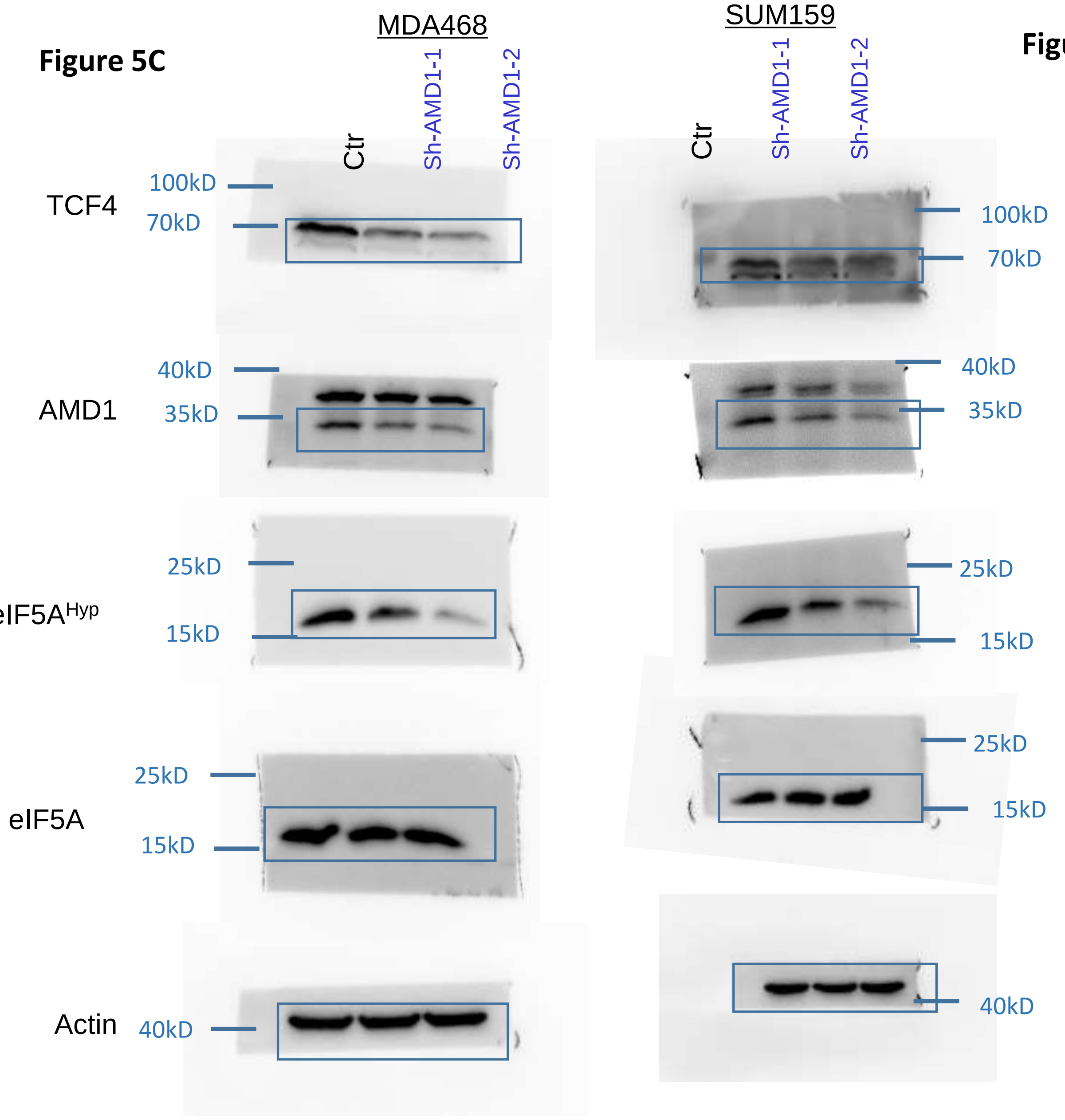

Figure 5D

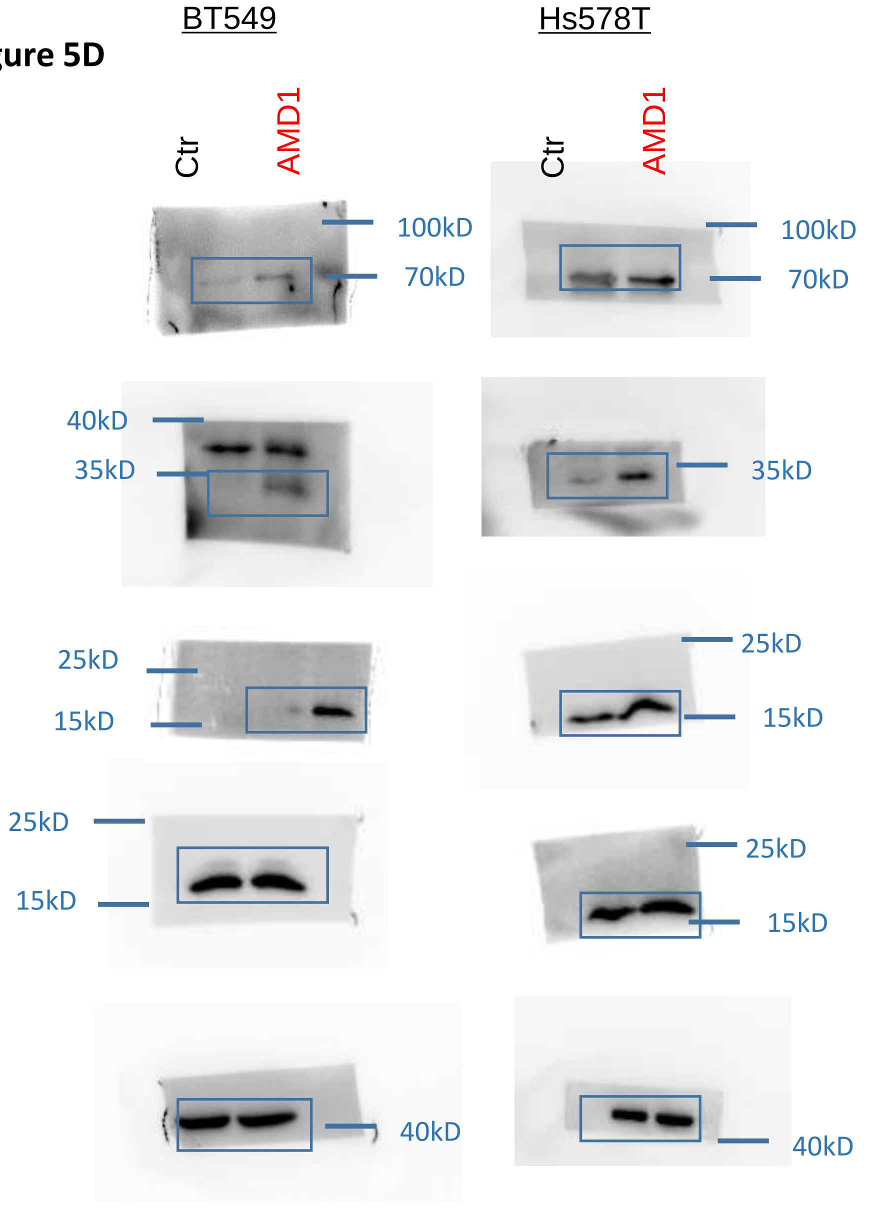

Figure 5E

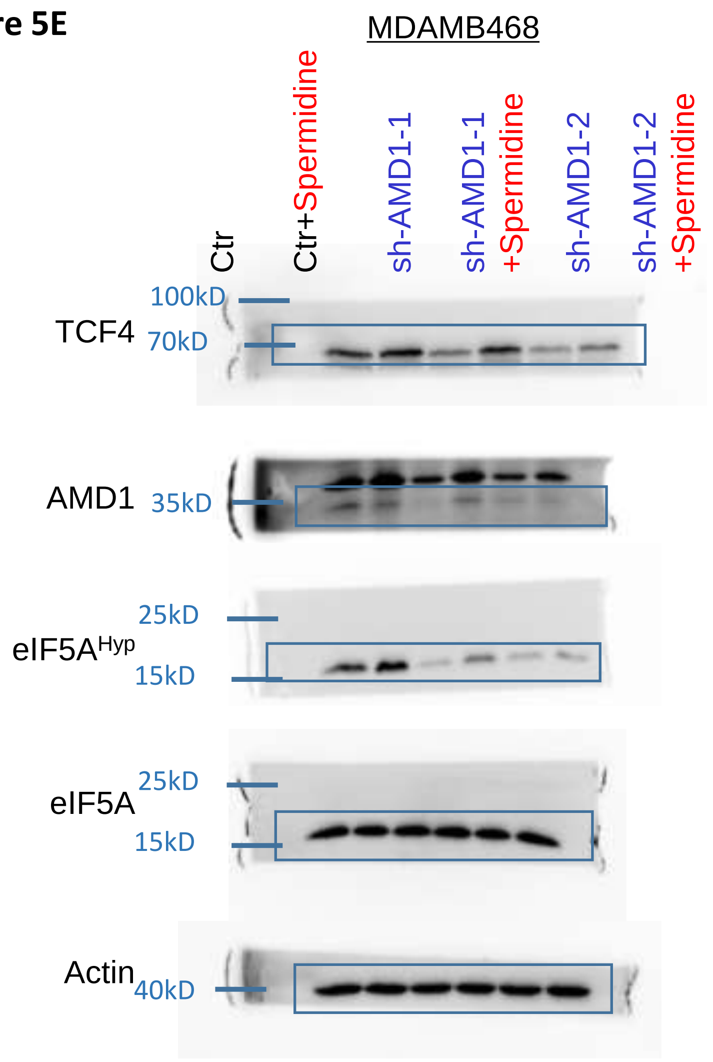

Figure 5F

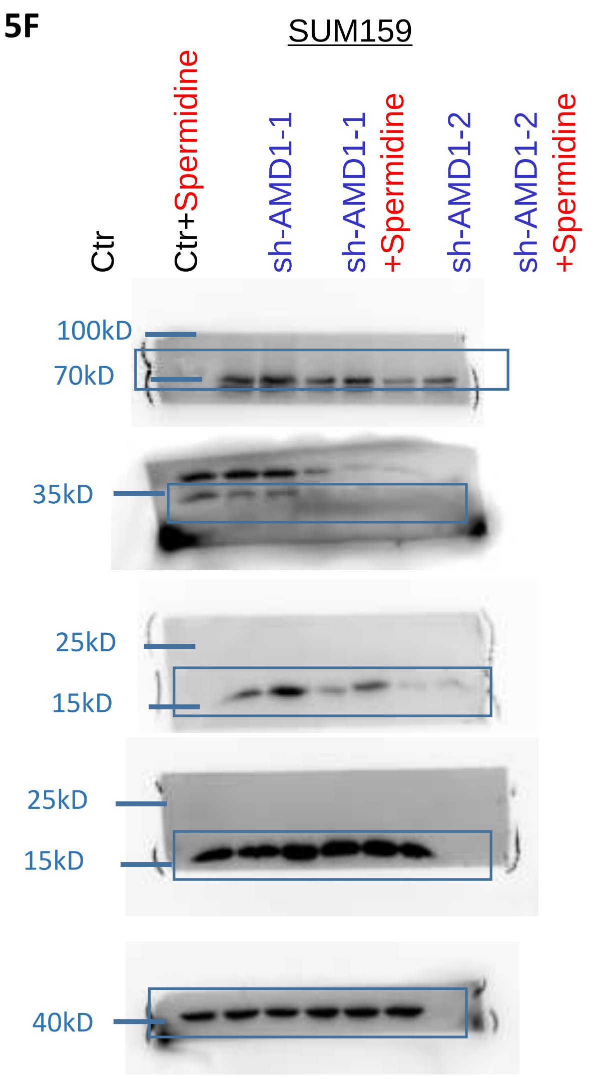

Figure 6B

Figure 6C

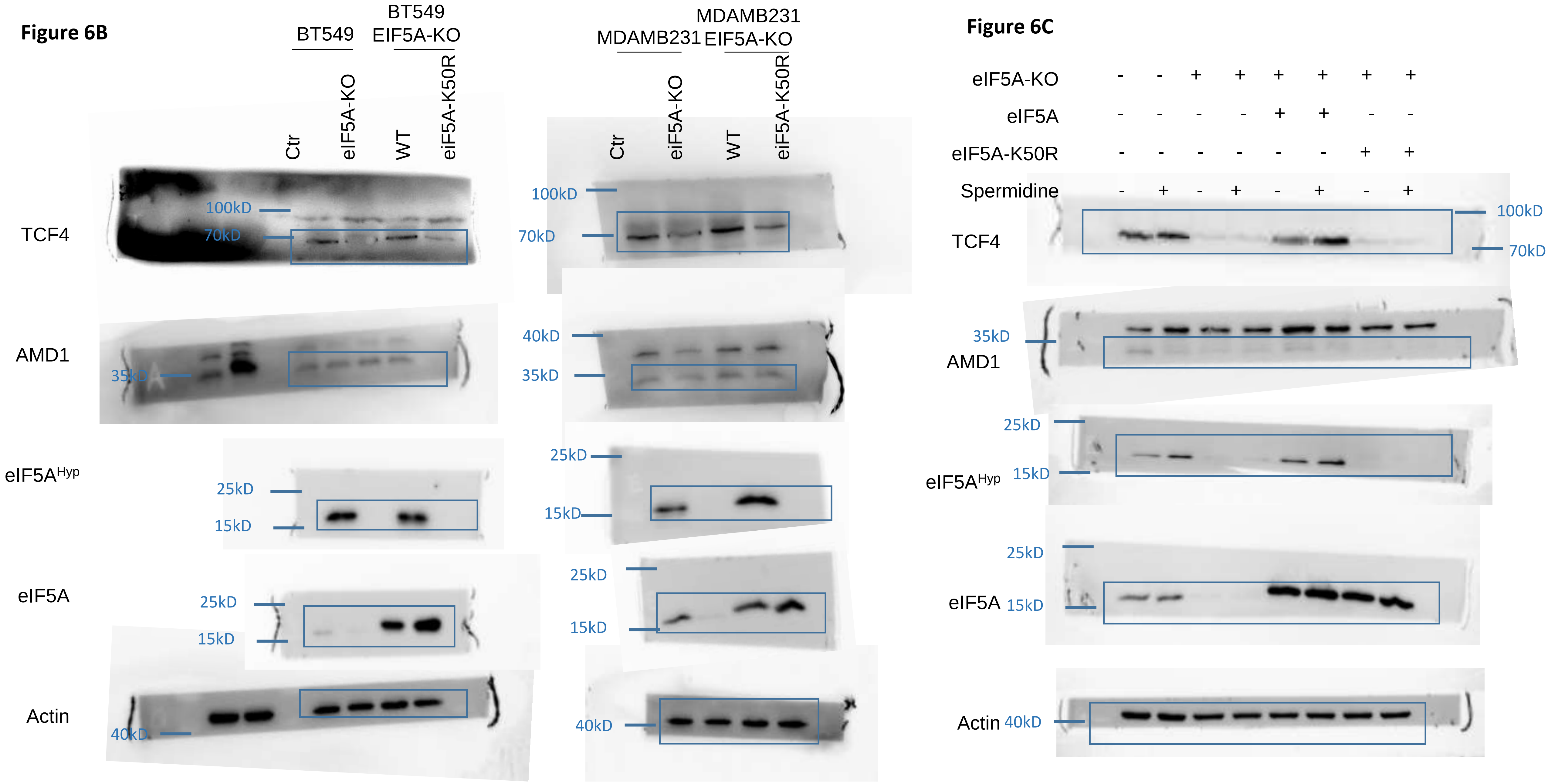

Figure 7E

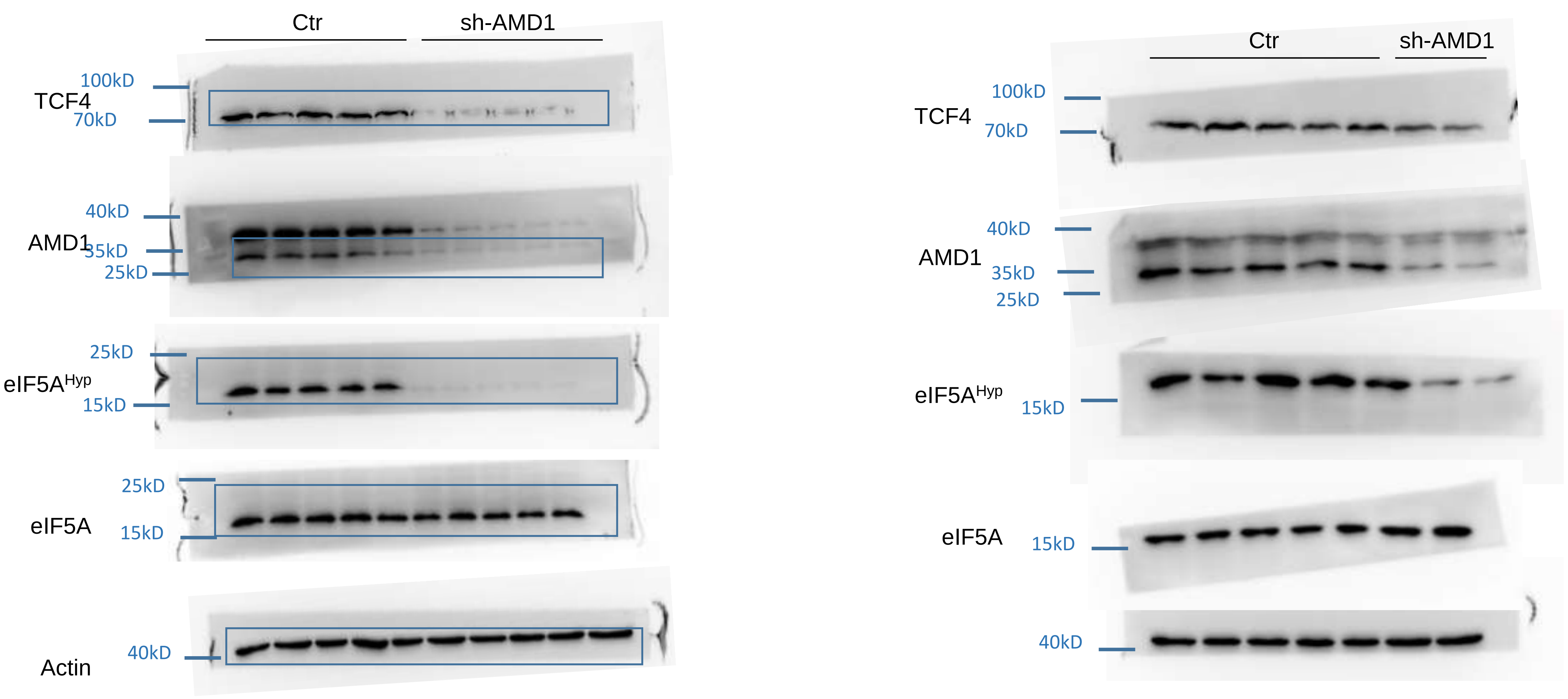
Ctr
sh-AMD1
TCF4
100kD
70kD
AMD1
40kD
35kD
25kD
eIF5AHyp
25kD
15kD
eIF5A
25kD
15kD
Actin
40kD
Ctr
sh-AMD1
TCF4
100kD
70kD
AMD1
40kD
35kD
25kD
eIF5AHyp
15kD
eIF5A
15kD
40kD

Figure S4A

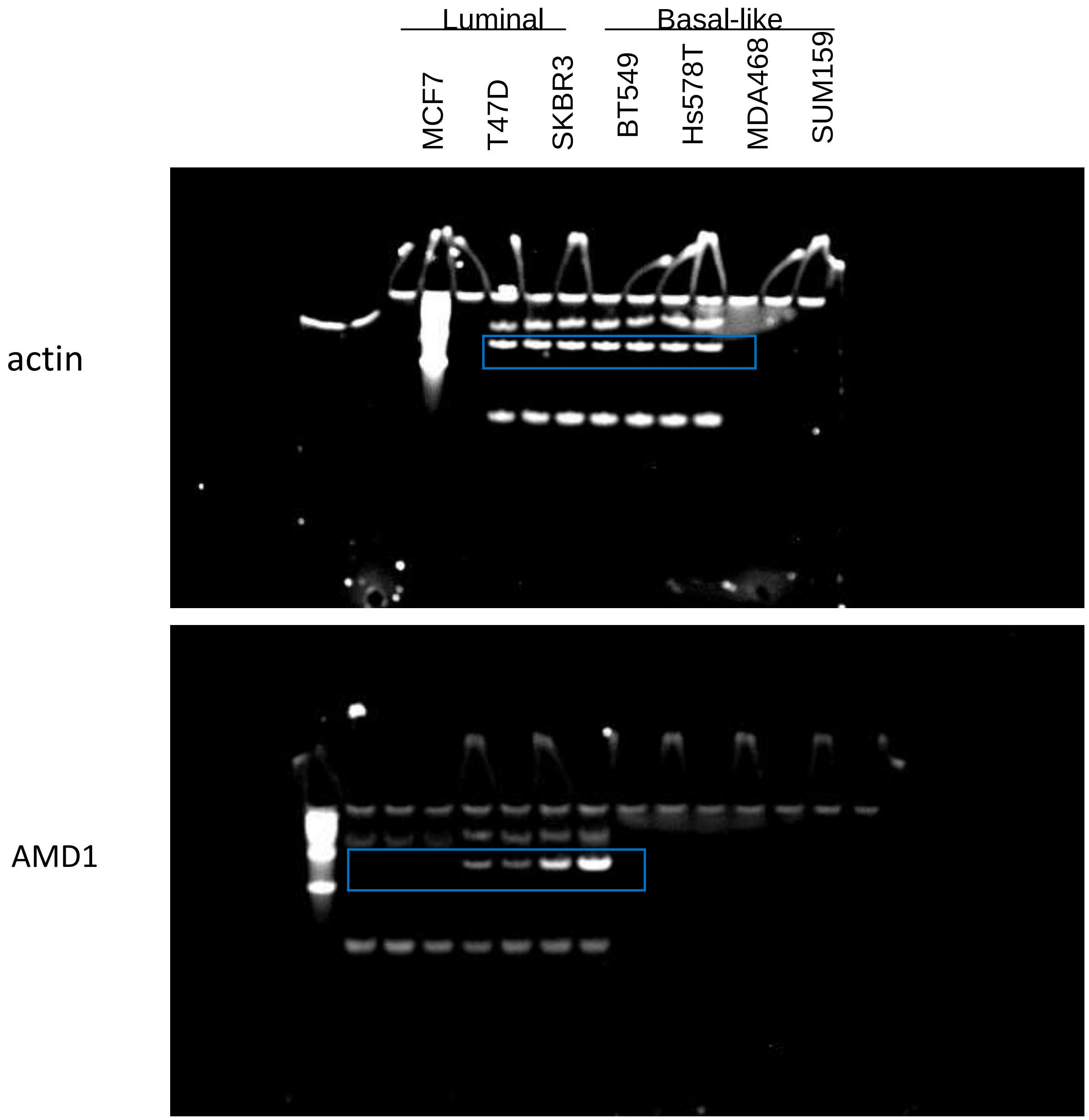
Luminal
Basal-like
MCF7
T47D
SKBR3
BT549
Hs578T
MDA468
SUM159
actin
AMD1
